# Cellular scaling rules for the brain of Artiodactyla include a highly folded cortex with few neurons

**DOI:** 10.3389/fnana.2014.00128

**Published:** 2014-11-12

**Authors:** Rodrigo S. Kazu, José Maldonado, Bruno Mota, Paul R. Manger, Suzana Herculano-Houzel

**Affiliations:** ^1^Instituto de Ciências Biomédicas, Universidade Federal do Rio de JaneiroRio de Janeiro, Brazil; ^2^Instituto Nacional de Neurociência Translacional, CNPq/MCT, SãoPaulo, Brazil; ^3^Microbrightfield Biosciences, BurlingtonVT, USA; ^4^Instituto de Física, Universidade Federal do Rio de JaneiroRio de Janeiro, Brazil; ^5^School of Anatomical Sciences, University of the WitwatersrandJohannesburg, South Africa

**Keywords:** evolution, cortical expansion, numbers of neurons, gyrification, brain size

## Abstract

Quantitative analysis of the cellular composition of rodent, primate, insectivore, and afrotherian brains has shown that non-neuronal scaling rules are similar across these mammalian orders that diverged about 95 million years ago, and therefore appear to be conserved in evolution, while neuronal scaling rules appear to be free to vary in a clade-specific manner. Here we analyze the cellular scaling rules that apply to the brain of artiodactyls, a group within the order Cetartiodactyla, believed to be a relatively recent radiation from the common Eutherian ancestor. We find that artiodactyls share non-neuronal scaling rules with all groups analyzed previously. Artiodactyls share with afrotherians and rodents, but not with primates, the neuronal scaling rules that apply to the cerebral cortex and cerebellum. The neuronal scaling rules that apply to the remaining brain areas are, however, distinct in artiodactyls. Importantly, we show that the folding index of the cerebral cortex scales with the number of neurons in the cerebral cortex in distinct fashions across artiodactyls, afrotherians, rodents, and primates, such that the artiodactyl cerebral cortex is more convoluted than primate cortices of similar numbers of neurons. Our findings suggest that the scaling rules found to be shared across modern afrotherians, glires, and artiodactyls applied to the common Eutherian ancestor, such as the relationship between the mass of the cerebral cortex as a whole and its number of neurons. In turn, the distribution of neurons along the surface of the cerebral cortex, which is related to its degree of gyrification, appears to be a clade-specific characteristic. If the neuronal scaling rules for artiodactyls extend to all cetartiodactyls, we predict that the large cerebral cortex of cetaceans will still have fewer neurons than the human cerebral cortex.

## INTRODUCTION

Artiodactyls are even-toed hoofed mammals, such as pigs, antelopes, and giraffes, of large body and brain size, with a highly convoluted cerebral cortex. Artiodactyls group together with cetaceans under the order Cetartiodactyla, which consists of 290 extant species (Wilson and Reeder, 1993). Despite the morphological distinctions between extant cetaceans and artiodactyls, the order Cetartiodactyla is evolutionarily monophyletic in origin, having diverged from the shared common ancestor with the nearest groups (Perissodactyla and Carnivora) around 90 million years ago (Murphy et al., 2004).

Artiodactyl brains are of great interest from the standpoint of comparative neuroanatomy, amongst other reasons for being comparable in size to the largest non-human primate brains. Despite this, few artiodactyl brains have been investigated so far: early studies of neuronal density in the cerebral cortex occasionally included cow (Tower and Elliott, 1952) and sheep (Haug, 1987), if they investigated artiodactyls at all, although their highly folded cerebral cortex drew attention already 40 years ago (Schlenska, 1974). Artiodactyl brains vary over 10 times in mass within a range that overlaps both with the largest non-human primates and with large carnivores, and the artiodactyl cerebral cortex is even more folded than that of primates and carnivores for a similar brain mass (Pillay and Manger, 2007; Manger et al., 2012). Yet, artiodactyls, primates, and carnivores of similar brain sizes have very distinct behavioral repertoires. While artiodactyls are mostly grazing herbivores, carnivores exhibit complex hunting behaviors, and primates are usually considered to exhibit an even more complex and flexible range of feeding and social behaviors than artiodactyls and carnivores (Roth and Dicke, 2005; Premack, 2007). Considering that the number of brain neurons must be a major limiting factor to computational capacity (Williams and Herrup, 1988), the similarity in brain size in the face of major behavioral differences raises the possibility that the brains of artiodactyls and primates of similar brain size do not contain similar numbers of neurons – that is, that they differ in their cellular composition, contrary to what was the dominant view in the past century (reviewed in Herculano-Houzel, 2011).

Using a novel method that allows the rapid quantification of the cellular composition of brain structures (the isotropic fractionator; Herculano-Houzel and Lent, 2005), we have previously shown that primates differ from rodents, lagomorphs (Glires, collectively), and afrotherians in the relationship between the mass of the cerebral cortex and the number of neurons that constitute it (Herculano-Houzel et al., 2006, 2007, 2011; Azevedo et al., 2009; Sarko et al., 2009; Gabi et al., 2010; Neves et al., 2014). Primates stand out in having a much larger number of neurons in the cerebral cortex than rodents of a similar cortical mass, as well as a larger number of neurons in the cerebellum than predicted for glires or afrotherians with a similar cerebellar mass (Herculano-Houzel, 2012; Neves et al., 2014). Given the behavioral differences between artiodactyls and primates, and assuming that neurons are the functional information-processing units of the brain, we predict artiodactyls to have fewer neurons than primates in both the cerebral cortex and cerebellum, given structures of a similar mass.

We refer to the mathematical relationship that describes how the mass of a brain structure varies as a function of the number of neurons in the structure across species as the *neuronal scaling rule* that applies to that structure. Similarly, the mathematical relationship that describes how the mass of a brain structure varies as a function of its number of non-neuronal cells across species is referred to as the *non-neuronal scaling rule* for that structure. The picture emerging from the isotropic fractionator studies is one where neuronal scaling rules are variable across brain structures and mammalian orders, while the non-neuronal scaling rules are shared across all brain structures and across all species of the different orders examined so far: Afrotheria, Glires, Scandentia, Primata, and Eulipotyphla (reviewed in Herculano-Houzel, 2011; Herculano-Houzel et al., 2014).

We have found that the neuronal scaling rules that apply to the cerebral cortex are shared amongst afrotherians, glires, and eulipotyphlans, but differ from those that apply to primates; the neuronal scaling rules that apply to the cerebellum are shared amongst afrotherians and glires, while eulipotyphlans and primates have their own separate scaling rules (Neves et al., 2014). In contrast, the neuronal scaling rules that apply to the “rest of brain” (brainstem plus diencephalon and basal ganglia) appear to be shared across afrotherians, glires, eulipotyphlans, and primates, although there is a larger spread of data points than for the other structures (Herculano-Houzel, 2011; Neves et al., 2014). These findings raise the possibility that, while mammalian orders may have characteristic neuronal scaling rules that apply to some brain structures, those neuronal scaling rules shared by afrotherians and glires, and also eulipotyphlans for the cerebellum and rest of brain, also applied to building the brains of the common mammalian ancestor to these modern groups (Herculano-Houzel et al., 2014; Neves et al., 2014).

Here we determine whether different neuronal scaling rules apply to Artiodactyla than to other mammalian orders, or whether artiodactyls have retained the neuronal scaling rules that we found to be shared by glires and afrotherians (Neves et al., 2014). We also examine whether non-neuronal scaling rules remained conserved across brain structures and species in the evolution of artiodactyls. We test these hypotheses by determining the cellular composition of the brain of five artiodactyl species of a wide range of brain sizes: the domestic pig, the springbok, the blesbok, the greater kudu, and the giraffe, by means of the isotropic fractionator (Herculano-Houzel and Lent, 2005), a non-stereological method that allows for the absolute numbers of neurons and non-neuronal cells to be readily quantified in anatomically defined regions of the brain. The isotropic fractionator yields results that are comparable to those obtained with stereology (Bahney and von Bartheld, 2014; Miller et al., 2014).

In addition to investigating how the cellular scaling rules for artiodactyls relate to previously found scaling rules, we compare the cellular composition of their brains specifically to those of primates, which have a similar range of brain sizes and cortical gyrification. We have previously shown that cortical folding scales differently across rodents and primates with numbers of cortical neurons (Ventura-Antunes et al., 2013), and proposed that folding is a consequence of tension related to connectivity through the white matter rather than a function of the simple addition of neurons to the cortical surface (Mota and Herculano-Houzel, 2012). Here we extend to the highly convoluted artiodactyl cerebral cortex our test of the hypotheses that the degree of cortical folding is (1) a similar function of whole brain mass (Hofman, 1985), (2) a shared function of the number of neurons in the cerebral cortex (reviewed in Mota and Herculano-Houzel, 2012), and (3) inversely correlated with cortical thickness (Hofman, 1985; Pillay and Manger, 2007). Finally, we examine whether variations in the degree of folding along the anteroposterior axis of the cerebral cortex are also related to local variations in the number of cortical neurons or in cortical thickness, and whether the artiodactyl cerebral cortex, like the human cerebral cortex, has separate zones that differ in their neuronal densities (Ribeiro et al., 2013).

## MATERIALS AND METHODS

### ANIMALS

One specimen each of domestic pig (*Sus scrofa domesticus*, 100 kg), blesbok (*Damaliscus dorcas phillipsi*, 60 kg), springbok (*Antidorcas marsupialis*, 25 kg), greater kudu (*Tragelaphus strepsiceros*, 218 kg), and giraffe (*Giraffa camelopardalis*, 470 kg) was used in the current study. The specimen of the domestic pig was obtained from local breeders in South Africa, whereas all other specimens were obtained from animals caught from field populations living in their natural environment, and thus their precise age could not be ascertained. All animals were healthy males with no obvious pathologies upon veterinary examination, and no neuropathologies visible, and had the typical body mass of adults, except for the giraffe, which was a juvenile (the typical adult body mass for giraffes is over 1,000 kg). All animals were treated and used in accordance with the University of the Witwatersrand Animal Ethics Committee Guidelines (clearance number 2008/36/1) which parallel those of the NIH for the care and use of animals in scientific experiments.

### DISSECTION

**FIGURE 1:**
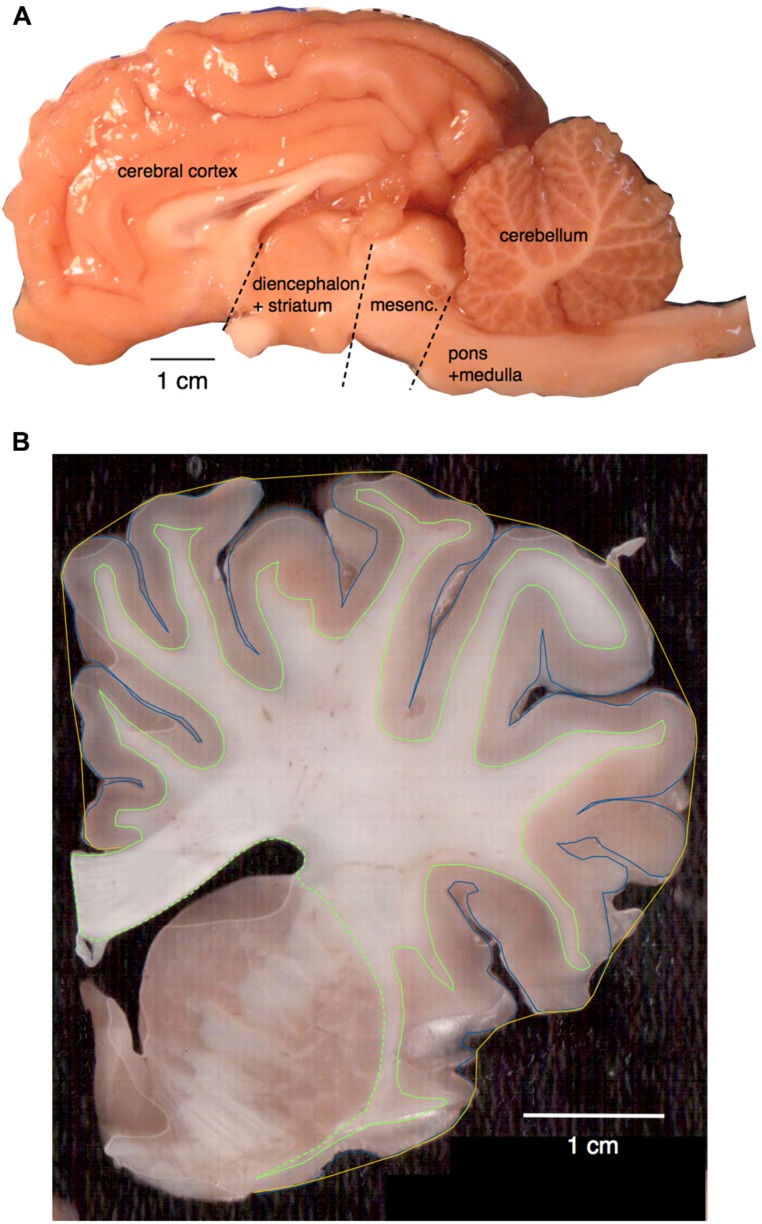
**(A)** Main structures analyzed. Shown is the hemisphere of *Damaliscus dorcas*, illustrating the separation of pons + medulla from the mesencephalon, and the cut that separated the latter from the diencephalon. The ensemble of diencephalon + striatum were later separated from the cerebrum in the individual coronal sections cut from the hemisphere already stripped of the mesencephalon. **(B)** Surface areas and volumes shown on a coronal section of *Giraffa camelopardalis*. Total pial surface perimeter (P_G_) is shown in blue; exposed perimeter, in yellow; perimeter of the white–gray matter interface (P_W_) in green (solid line); and area of subcortical white matter in the coronal plane also in green, including the dashed line.

All animals were euthanized (overdose of sodium pentobarbital, 100 mg/kg, i.v.) and the head was perfused through the carotids with 0.9% saline, followed by 4% paraformaldehyde in 0.1 M phosphate buffer (PB, pH 7.4). Following perfusion, the brains were removed, weighed, and post-fixed in 4% paraformaldehyde in 0.1 M PB overnight, cryoprotected in 30% sucrose in 0.1 M PB at 4∘C and stored in an antifreeze solution at -20∘C until processing (Manger et al., 2009). The brains were divided into two halves along the mid-sagittal fissure and one hemisphere of each brain processed. The olfactory bulbs, when available, were dissected and weighed individually. The cerebellum was dissected by cutting the cerebellar peduncles at the surface of the brainstem. The cerebrum was separated from the brainstem by cutting at a plane anterior to the colliculi and posterior to the thalamus and mammillary bodies of the hypothalamus. The brainstem was divided into pons + medulla and mesencephalon by an axial transection anterior to the basilar pons and posterior to the inferior colliculus (**Figure 1A**). The cerebrum was then cut manually into 2 mm coronal sections in order to allow the removal of the ensemble of diencephalon and striatum, of the hippocampus, and the dissection of the remaining cerebral cortex into gray and white matter, which had their numbers of cells counted separately. Numbers of cells obtained separately for the pons + medulla, mesencephalon, and diencephalon + striatum were later pooled together and are reported as “rest of brain,” for comparison with data obtained previously in other species (Herculano-Houzel et al., 2006, 2007, 2011; Azevedo et al., 2009; Sarko et al., 2009; Gabi et al., 2010; Neves et al., 2014).

Coronal sections of the cerebral cortex were pooled in groups of three (amounting to 6 mm sections) for analysis of the distribution of neurons along the anterior–posterior axis. Each set of coronal sections was then dissected into gray and white matter prior to cell counting. This procedure was followed in all species except for the blesbok, whose cerebral cortex was processed separately as gray and white matter only.

Since only one hemisphere of each brain was available for analysis, values reported here are multiplied by 2 to give estimates for the whole brain that are comparable with our previously published data (Herculano-Houzel et al., 2006, 2007, 2011; Sarko et al., 2009; Gabi et al., 2010; Neves et al., 2014). While this ignores possible asymmetries between the hemispheres, such asymmetries would have no influence on the results reported here, as the latter span several orders of magnitude whereas any asymmetries would be of the order of a few percentual points between the hemispheres. For the sake of consistency with our previous studies, and because the olfactory bulb was not available for all specimens, whole brain values used in the analysis exclude the olfactory bulb.

### MORPHOMETRY

All coronal sections of the cerebral cortex were scanned at 300 dpi and imported into NeuroLucida software (MBF Bioscience, Vermont, USA) for tracing and reconstruction of cortical gray and white matter surface areas and volumes, as described previously (Ribeiro et al., 2013). Briefly, for each 2 mm section, we determined the total pial surface perimeter (P_G_) and the area of gray matter in the coronal plane (S_G_); the perimeter of the white–gray matter interface (P_W_, which does not include the interface with the striatum); and the area of subcortical white matter external to the striatum in the coronal plane (S_W_; **Figure 1B**).

Determining cortical surface area and volumes by multiplying coronal perimeters and areas by section thickness (2 mm) would underestimate areas and volumes at both extremities of the cerebral cortex, due to its curved shape. Instead, we used formulas that calculate area and volume from a series of sections in a manner that is sensitive to the inclination of the cerebral cortical surface (Ribeiro et al., 2013). These formulas are based on the measurements of the gray or white matter perimeters in the coronal plane, P, and of the coronal area occupied by the gray or white matter in the section, S. The formulas that calculate total gray and white matter surface areas A_G_ and A_W_ and volumes V_G_ and V_W_ for each section in a series of coronal areas and perimeters S_0_, S_1_, S_2_, ... and P_0_, P_1_, P_2_, ... obtained as above are the following:

$$A_{n}={\left\{{\left(S_{n}--S_{n-1}\right)}^{2}+{\left[h\left(P_{n}+P_{n-1}\right)/2\right]}^{2}\right\}}^{1/2}$$

$$V_{n}=h\left[S_{n}+S_{n-1}+{\left(S_{n}\cdot S_{n-1}\right)}^{1/2}\right]/3$$

where h = 2 mm (section thickness).

We calculated the local cortical thickness (T) of the gray matter as the ratio V_G_/A_G_. The local folding index (FI), a measure of the degree of cortical gyrification of a coronal section (Zilles et al., 1988), was calculated by first tracing the exposed surface of the gray matter (the smallest surface of gray matter that did not enter sulci) to determine A_E_ in a section, then determining the ratio A_G_/A_E_ for that section. The total FI for each species is the average of the folding indices for all sections.

After scanning, each section had its cortical white and gray matter separated with a scalpel, using the small amount of resistance that the white matter offers to being pulled from the gray matter exactly at the gray/white matter interface. The hippocampus was removed from all sections. We recorded the mass (g) of the gray and white matter of each analyzed structure to determine cell densities in a manner that is comparable with our previous studies. Sections were then pooled in groups of three for quantification of their cellular composition. Surface areas and volumes were correspondingly added across the groups of three sections for analysis. To determine the total number of neuronal and other (mostly glial) cells in each structure, we then applied the Isotropic Fractionator (Herculano-Houzel and Lent, 2005), which transforms the anisotropic cerebral tissue into an isotropic suspension of cell nuclei whose density in the suspension of known volume can promptly be determined.

### ISOTROPIC FRACTIONATION

Total numbers of cells, neurons, and non-neuronal (other cells) were estimated as described previously using the isotropic fractionator method (Herculano-Houzel and Lent, 2005). Briefly, this method turns each dissected brain division into an isotropic suspension of isolated nuclei of known, defined volume, kept homogeneous by agitation. The total number of nuclei in suspension – and therefore the total number of cells in the original tissue – is estimated by determining the density of nuclei in small aliquots stained with the fluorescent DNA marker DAPI (4′-6-diamidino-2-phenylindole dihydrochloride, Invitrogen, USA) under the microscope.

For each structure, at least four samples of the nuclear suspension were counted independently, in different chambers of the hemocytometer, to determine the number of nuclei/ml of the suspension. The reported values for total number of cells refer to the average nuclei/ml of the samples taken multiplied by the total volume of the suspension. This consistently yields a coefficient of variation of 0.10 or less, and never more than 0.15, across samples from the same structure.

Once the total cell number in a structure is known, the proportion of neurons is determined by immunocytochemical detection of neuronal nuclear antigen (NeuN), expressed in the nuclei of most neuronal cell types and not in non-neuronal cells (Mullen et al., 1992; Gittins and Harrison, 2004). We used a polyclonal primary antibody against NeuN that is labeled with Cy3 (Millipore, ABN78), which gives results similar to those obtained with the unlabeled monoclonal primary (Millipore, MAB377) followed by a secondary antibody reaction, but yields a much stronger signal in a single step reaction. Estimates of the proportion of NeuN-positive nuclei are considered reliable since the coefficient of variation among animals of the same species is typically below 0.15. Numbers of other cells are derived by subtraction.

### DATA ANALYSIS

All statistical analyses and regressions were performed in JMP 9.0 (SAS Institute, Cary, NC, USA). Regressions to power and linear functions were performed to find the best fit for each distribution.

For the comparison with cellular scaling rules reported previously, we used the equations that apply to the average structure size and cellular composition for the species of the groups described earlier: Primata (Gabi et al., 2010), excluding tree shrews; Glires (Herculano-Houzel et al., 2006, 2011; excluding the naked mole-rat, the only fossorial animal in our sample, which is an outlier amongst Glires – see Herculano-Houzel et al., 2011), Eulipotyphla (Sarko et al., 2009), and Afrotheria (Neves et al., 2014). Because we found the artiodactyl data points to overlap with the distributions for glires and afrotherians, we also tested their alignment with the power function that applies jointly to glires and afrotherians.

The hand dissection of the cerebral cortex into gray and white matter yields small numbers of neurons in the “white matter.” Checks performed in cores of white matter dissected with no risk of contaminating gray matter yielded no significant numbers of neurons in the tissue, which suggests that neurons found in white matter preparations are contaminants from the gray matter, and not resident neurons in the white matter, although interstitial neurons are known to exist in other species (Chun and Shatz, 1989). For consistency, all analyses that refer to scaling with cortical surface area or volume use total numbers of neurons (gray and white combined), except values reported explicitly for the gray matter, such as neuronal density in the gray matter, which compute only the numbers of neurons found in the dissected tissue (**Table 1**).

**Table 1:** Cellular composition of Artiodactyla brains.

	*Sus scrofa domesticus*	*Antidorcas marsupialis*	*Damaliscus dorcas phillipsi*	*Tragelaphus stripceros*	*Giraffa camelopardalis*
M_BD_, kg	∼100	25	60	218	470
M_BR_, g	57.758	102.102	155.066	308.522	528.026
M_CXT_, g	35.780	62.814	109.044	212.004	389.616
M_HP_, g	1.928	3.434	n.a.	10.936	7.486
M_CB_, g	8.128	11.458	13.402	31.776	67.730
M_RoB_, g	13.850	27.830	32.620	64.742	70.680
M_D__+__BG_, g	6.728	13.814	n.a.	30.418	33.322
M_MES_, g	2.338	5.304	n.a.	15.928	15.928
M_P__+__M_, g	4.784	8.312	n.a.	18.396	21.430
M_OB_, g	0.822	1.200	n.a.	5.546	2.052
N_BR_	2.22 × 10^9^	3.06 × 10^9^	3.90 × 10^9^	6.09 × 10^9^	11.21 × 10^9^
N_CXT_	292.96 × 10^6^	375.49 × 10^6^	548.58 × 10^6^	793.07 × 10^6^	1.67 × 10^9^
N_GM_	207.75 × 10^6^	293.77 × 10^6^	361.95 × 10^6^	615.87 × 10^6^	1.33 × 10^9^
N_HP_	12.91 × 10^6^	20.48 × 10^6^	n.a.	28.36 × 10^6^	58.59 × 10^6^
N_CB_	1.86 × 10^9^	2.26 × 10^9^	2.40 × 10^9^	4.04 × 10^9^	8.88 × 10^9^
N_RoB_	58.71 × 10^6^	70.48 × 10^6^	92.24 × 10^6^	106.28 × 10^6^	150.65 × 10^6^
N_D+BG_	34.40 × 10^6^	40.12 × 10^6^	n.a.	58.88 × 10^6^	34.32 × 10^6^
N_MES_	12.43 × 10^6^	7.52 × 10^6^	n.a.	26.07 × 10^6^	26.63 × 10^6^
N_P__+__M_	11.88 × 10^6^	22.84 × 10^6^	n.a.	21.64 × 10^6^	47.44 × 10^6^
N_OB_	9.20 × 10^6^	16.00 × 10^6^	58.71 × 10^6^	38.33 × 10^6^	24.68 × 10^6^
DN_CXT_	8,118	5,978	5,031	3,741	4,283
DN_GM_	7,375	7,051	5,142	4,798	5,882
DN_HP_	6,695	5,965	n.a.	2,594	7,826
DN_CB_	228,632	196,999	179,206	127,218	131,080
DN_RoB_	4,238	2,532	2,828	1,642	2,131
DN_D__+__BG_	5,113	2,904	n.a.	1,936	1,030
DN_MES_	5,317	1,418	n.a.	1,637	1,672
DN_P__+__M_	2,483	2,748	n.a.	1,176	2,214
DN_OB_	11,187	13,332	n.a.	6,912	12,026
O/N_BR_	2.076	3.251	3.989	2.481	3.252
O/N_CXT_	10.361	10.834	11.956	14.625	15.949
O/N_GM_	8.544	7.239	8.356	8.443	7.764
O/N_HP_	10.334	10.111	n.a.	17.868	10.628
O/N_CB_	0.188	0.207	0.184	0.313	0.622
O/N_RoB_	18.682	18.710	24.842	32.076	32.333
O/N_D__+__BG_	17.779	19.408	n.a.	31.841	38.841
O/N_MES_	15.667	30.250	n.a.	30.546	41.017
O/N_P__+__M_	24.452	13.706	n.a.	34.088	23.631
O/N_OB_	8.434	6.576	n.a.	8.523	9.417

*Cellular composition of the five artiodactyl species. M, mass of body (M_BD_) or brain structure; N, number of neurons; DN, neuronal density (in neurons/mg); O/N, ratio between numbers of other (non-neuronal) cells and neurons. BR, whole brain (excluding the olfactory bulb); CXT, whole cerebral cortex (gray matter, white matter, and hippocampus); HP, hippocampus; CB, cerebellum; RoB, rest of brain (the sum of diencephalon + basal ganglia, mesencephalon, and pons + medulla); D + BG, diencephalon + basal ganglia; MES, mesencephalon; P + M, pons + medulla; OB, olfactory bulb. All values refer to the two hemispheres together.*

## RESULTS

**FIGURE 2:**
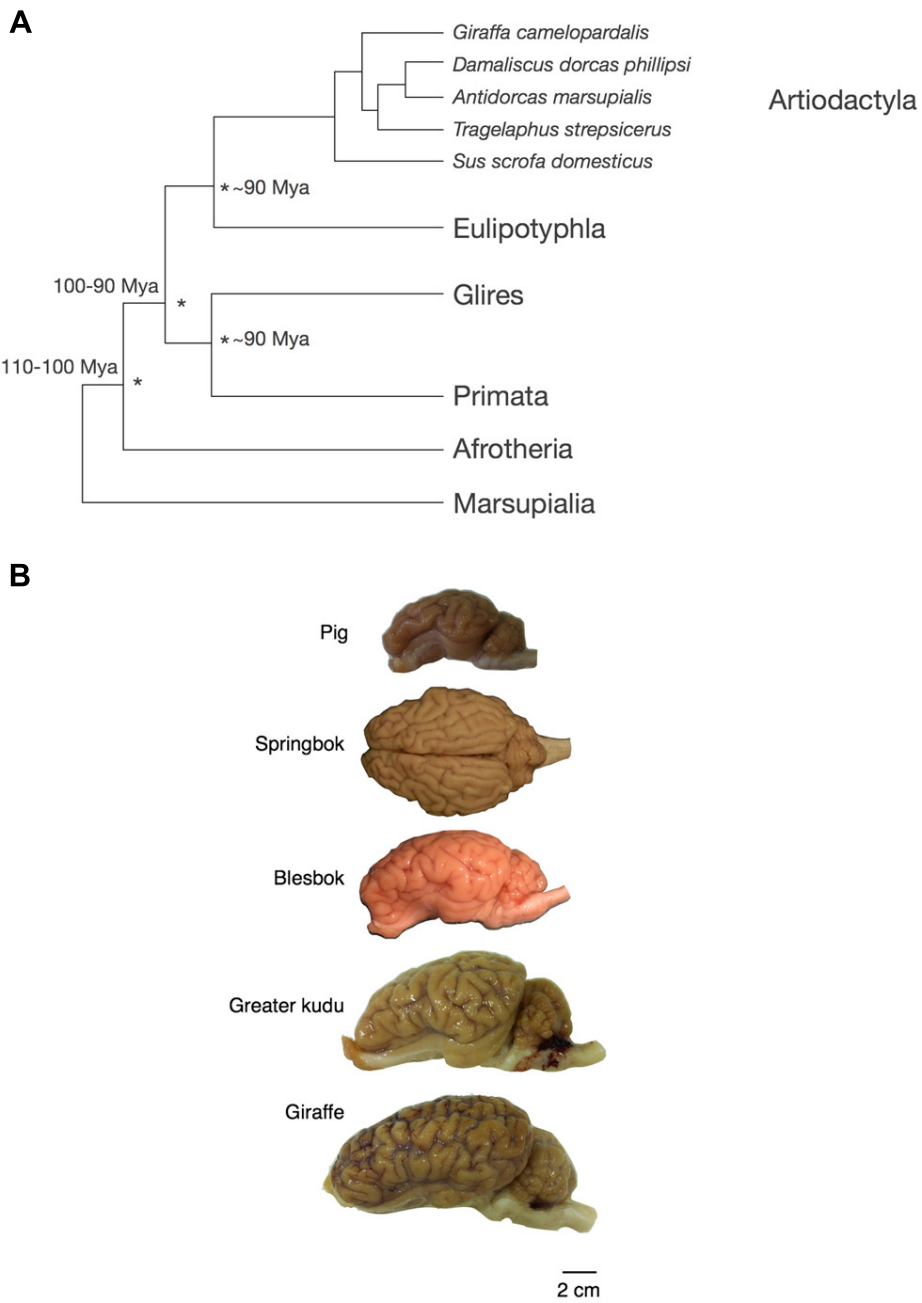
**Phylogenetic relationship and appearance of the artiodactyl brains used in this study. (A)** Phylogenetic relationship across the artiodactyl species examined here and other mammals investigated previously. **(B)** Appearance of the artiodactyl brains used in this study. Scale bar, 2 cm. The springbok brain is shown in a dorsal view, while the lateral view of the left hemisphere is shown for the other species.

**FIGURE 3:**
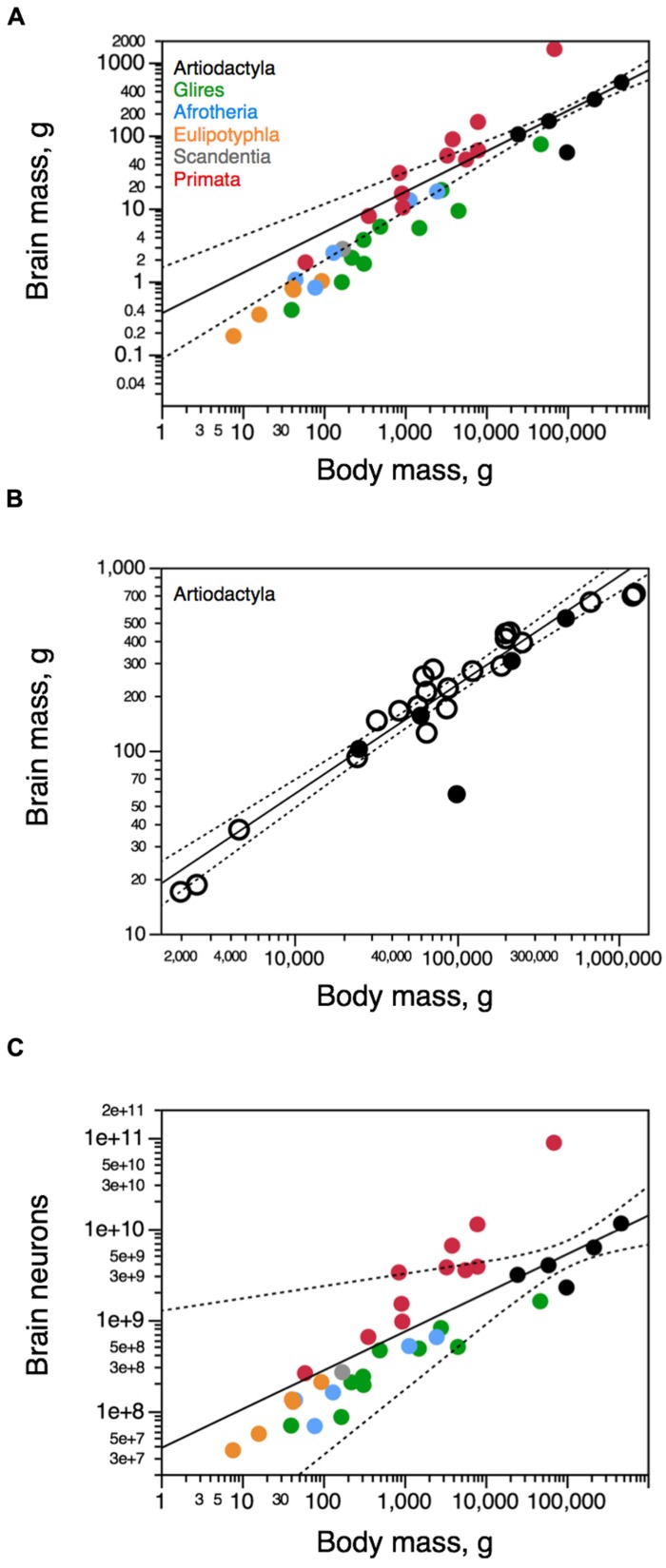
**Artiodactyl brains gain mass with a different relationship to body mass compared to other mammals.** Scaling relationships for artiodactyl brains (in black) are plotted along with rules found previously for other groups (primates in red, glires in green, afrotherians in blue, insectivores in orange, scandentia in gray). Graphs relate **(A,B)** total brain mass (excluding the olfactory bulb) and **(C)** total numbers of neurons in the brain (excluding the olfactory bulb) to body mass. **(A,C)** our dataset; **(B)** our dataset (filled circles), and dataset of Boddy et al. (2012; open circles). Each point represents the average values for each species. Only the power functions for Artiodactyla are plotted (excluding the pig), along with the 95% confidence interval (dotted line). Exponents are 0.555 ± 0.029 (**A**, brain mass × body mass), 0.596 ± 0.031 **(B**, brain mass × body mass, for the Boddy dataset exclusively), and 0.425 ± 0.069 (brain neurons × body mass). Data from Herculano-Houzel et al. (2006, 2007, 2011), Azevedo et al. (2009), Sarko et al. (2009), Gabi et al. (2010), and Neves et al. (2014).

Across the five species analyzed here (**Figure 2**), average body mass varies 18.8-fold (from 25 kg in the springbok to 470 kg in the giraffe), while brain mass varies 9.1-fold, and total number of brain neurons varies only 5.0-fold (**Table 1**). Brain mass increases as a power function of body mass with a small exponent of 0.555 ± 0.029 (*p* = 0.0027; **Figure 3A**, excluding the pig, which is an obvious outlier in the relationship, with a much larger body than expected for its brain mass, a probable consequence of domestication – see Figure 1 in Kruska, 2007). The relationship between brain mass and body mass for artiodactyls does not overlap with any of those found previously for glires, insectivores, afrotherians, or primates (**Figure 3A**), but it does overlap with the relationship found for an independent dataset of 22 artiodactyl species (Boddy et al., 2012), with a similarly small exponent of 0.596 ± 0.031 (*p* < 0.0001, **Figure 3B**), where again the domestic pig is an obvious outlier. Although the giraffe individual we analyze was a juvenile, with a smaller body and brain mass than in adult individuals, its body and brain mass still fit the relationship found in the dataset of Boddy et al. (2012; **Figure 3B**). The total number of brain neurons (not including the olfactory bulb) also increases as a power function of body mass, with an even smaller exponent of 0.425 ± 0.069 (*p* = 0.00254; **Figure 3C**), and this relationship also does not overlap with that found previously for other mammalian clades, although the wider 95% CI includes most other species analyzed previously (**Figure 3C**).

### RELATIVE DISTRIBUTION OF MASS AND NEURONS

On average, we find that the cerebral cortex as a whole (with the hippocampus) corresponds to 67.2 ± 2.4% of brain mass, the cerebellum amounts to 11.4 ± 0.9% of brain mass, and the rest of the brain to 21.3 ± 2.3% of brain mass, values in the same range as found for other mammals by ourselves (reviewed in Herculano-Houzel, 2011) and others (Clark et al., 2001). The relative size of the cerebral cortex and of the cerebellum (expressed as a percentage of total brain mass) does not vary significantly with total brain mass across species studied (Spearman correlation, *p* = 0.1041 and 0.6238, respectively), while the relative mass of the rest of brain decreases significantly with increasing brain mass (Spearman correlation, ρ = -0.900, *p* = 0.0374).

The relative number of brain neurons found in each structure does not vary significantly with brain mass across the five artiodactyls (all correlations, *p* > 0.18). As in other mammalian species, the cerebellum of these artiodactyls concentrates most of the brain neurons, with on average 82.3 ± 0.9% of all brain neurons despite its much smaller relative mass of 11.4 ± 0.9%. Conversely, the cerebral cortex has only 15.4 ± 0.8% of all brain neurons, despite representing 67.2 ± 2.4% of brain mass, and the rest of the brain, which accounts for 21.3 ± 2.3% of brain mass, has only 2.4 ± 0.3% of all brain neurons. Across species, variations in the relative mass of the cerebral cortex do not correlate with variations in relative number of brain neurons in the cerebral cortex (*p* = 0.2848), nor does a larger relative mass of the rest of brain correlate with a larger relative number of brain neurons in the rest of brain (*p* = 0.2848), although relatively larger cerebella contain relatively more of brain neurons (ρ = 0.900, *p* = 0.0374). The hippocampus is relatively small in artiodactyls, representing between 1.9% (in the giraffe) and 5.5% (in the springbok) of total cortical mass, and containing only between 3.5% (in the giraffe) and 5.4% (in the springbok) of all cortical neurons (**Table 1**).

### NEURONAL SCALING RULES

**FIGURE 4:**
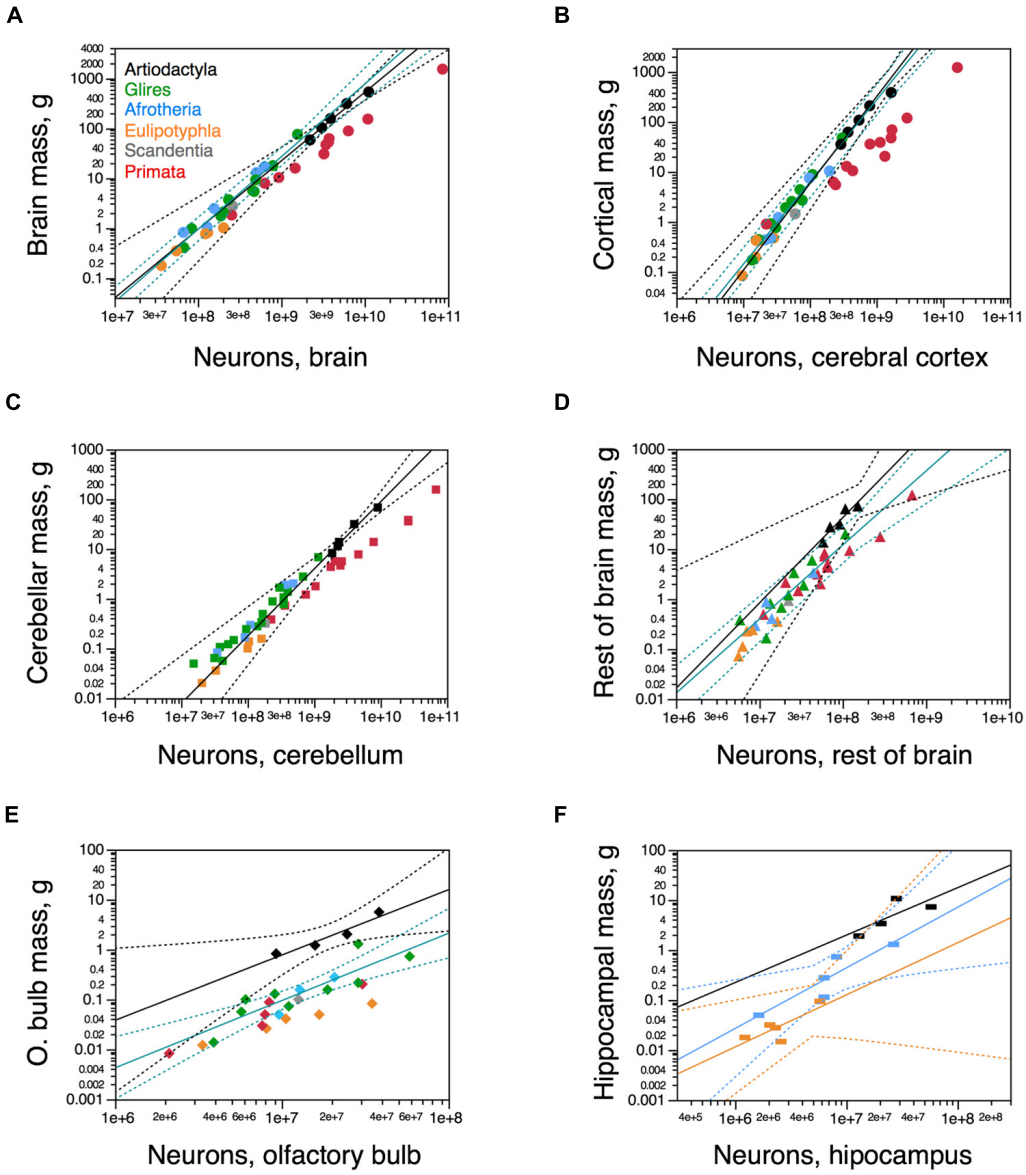
**Neuronal scaling rules for artiodactyl brains and brain structures.** Average brain structure mass for each species is plotted as a function of its total number of neurons. **(A)** Whole brain (excluding olfactory bulb); **(B)** Cerebral cortex (including hippocampus); **(C)** cerebellum; **(D)** rest of brain; **(E)** olfactory bulb; **(F)** hippocampus. **(A)** Artiodactyls overlap with the power function relating brain mass and number of brain neurons for glires and afrotherians together (green line; exponent, 1.456 ± 0.092), and the power function that applies to artiodactyls (black line, with the giraffe; exponent, 1.373 ± 0.120) also includes glires and afrotherians. **(B)** Artiodactyls overlap with the power function relating the mass of the cerebral cortex (including the hippocampus) and number of cortical neurons for glires and afrotherians together (green line; exponent, 1.645 ± 0.090), and the power function that applies to artiodactyls (black line, excluding the giraffe; exponent, 1.739 ± 0.092) also includes glires, afrotherians, and eulipotyphlans, but excludes primates.** (C)** The power function relating cerebellar mass and number of cerebellar neurons for artiodactyls (black line; exponent, 1.351 ± 0.118) includes other non-primate mammalian clades. **(D)** The power function relating mass and number of neurons in the rest of brain mass (brainstem to basal ganglia) for glires and afrotherians (green line; exponent, 1.481 ± 0.180) excludes four of five artiodactyl species. While all but one non-artiodactyl species fall below the power function that applies to artiodactyls (black line; exponent, 1.706 ± 0.376), the 95% CI is wide enough that they are still included. **(E)** The power function relating mass and number of neurons in the olfactory bulb for artiodactyls (black line; exponent, 1.309 ± 0.257) excludes all other mammalian clades. **(F)** The artiodactyl hippocampus falls within the 95% CI of the power functions relating hippocampal mass and number of neurons for afrotherians (blue line; exponent, 1.211 ± 0.312, *p* = 0.0304) and for eulipotyphlans (yellow line; exponent, 1.041 ± 0.422; *p* = 0.0903). Artiodactyl species in black, primates in red, glires in green, afrotherians in blue, insectivores in orange, scandentia in gray. Data from Herculano-Houzel et al. (2006, 2007, 2011), Azevedo et al. (2009), Sarko et al. (2009), Gabi et al. (2010), and Neves et al. (2014).

We find that the relationship between brain size and number of neurons is shared between artiodactyls, glires, and afrotherians, such that brains of a similar mass across these orders, if they existed, would have a similar number of neurons (**Figure 4A**). Across these five artiodactyls, total brain mass varies as a power function of its number of neurons with an exponent of 1.373 ± 0.120 (*r*^2^ = 0.977, *p* = 0.0015), indicating that the brain as a whole gains mass more rapidly than it gains neurons (**Figure 4A**). The distribution of brain mass and total number of neurons in artiodactyls falls within the 95% confidence interval of the joint distribution for glires (rodents and lagomorphs) and afrotherians, which, moreover, has a similar exponent of 1.456 ± 0.092 (*r*^2^ = 0.950, *p* < 0.0001; **Figure 4A**). In contrast, artiodactyls have much larger brains than primates with similar numbers of neurons (**Figure 4A**, compare black and red points).

The relationship between the mass of the cerebral cortex and its number of neurons for the five artiodactyl species has an exponent of 1.364 ± 0.150 (*p* = 0.0028). This, however, includes the giraffe, which was a juvenile, with a cerebral cortex that was possibly not yet fully grown, thus artificially decreasing the exponent (notice that Boddy et al. (2012) report a total brain mass of 700 g for the giraffe, while our specimen had a cortical mass of only 528 g). When the giraffe is excluded from the relationship, the exponent that applies to the cerebral cortex of artiodactyls (1.739 ± 0.092, *r*^2^ = 0.994, *p* = 0.0028; **Figure 4B**, black) becomes indistinguishable from the exponent that applies to this distribution across glires and afrotherians together, of 1.645 ± 0.090 (*r*^2^ = 0.962, *p* < 0.0001; **Figure 4B**, green). The mass of the cerebral cortex of artiodactyls is therefore well predicted from its number of neurons by the neuronal scaling rules that apply to glires and afrotherians. In contrast, the artiodactyl cerebral cortex is much larger than a primate cerebral cortex of a similar number of neurons (**Figure 4B**, compare black and red data points). Thus, the relationship between cerebral cortical mass and number of neurons that applies to glires and afrotherians also applies to artiodactyls, and to eulipotyphlans as well, but excludes the primates (**Figure 4B**, red data points).

The relationship between cerebellar mass and number of neurons across the five artiodactyl species is described by a power function with exponent 1.351 ± 0.118 (*r*^2^ = 0.978, *p* = 0.0014) that overlaps with the distribution that applies to glires and afrotherians, of exponent 1.306 ± 0.070 (*r*^2^ = 0.964, *p* < 0.0001) and includes data points for those species (**Figure 4C**). This relationship is such that, for a similar number of cerebellar neurons, artiodactyls have a much larger cerebellar mass than primates (**Figure 4C**).

The mass of the rest of brain scales as a power function of its number of neurons across artiodactyls with an exponent of 1.706 ± 0.376 (*r*^2^ = 0.872, *p* = 0.0201; **Figure 4D**, black). Although this exponent is not significantly different from the exponent that applies jointly to afrotherians and glires, of 1.481 ± 0.180 (*r*^2^ = 0.838, *p* < 0.0001; **Figure 4D**, green), four of the five artiodactyl data points fall outside of the 95% confidence interval for afrotherians and glires. This suggests that a different neuronal scaling rule applies to artiodactyls than to afrotherians and glires, and also to primates, such that the mass of the rest of brain is larger in artiodactyls than in afrotherians, glires, and also primates, for a similar number of neurons in the rest of brain. Within the rest of brain, we find larger numbers of neurons in the combined diencephalon and basal ganglia than in the mesencephalon or the combined pons and medulla in the smaller artiodactyls (pig, springbok, and kudu), but not in the giraffe (**Table 1**).

The mass of the olfactory bulb scales across artiodactyls as a function of its number of neurons of exponent 1.309 ± 0.257 (*r*^2^ = 0.928, *p* = 0.0364), similar to the exponent that applies to afrotherians and glires together (1.348 ± 0.238, *r*^2^ = 0.762, *p* = 0.0002; **Figure 4E**). However, the two distributions are shifted, so that the olfactory bulb of artiodactyls has a significantly larger mass than the olfactory bulb of afrotherians and glires with a similar number of neurons, falling outside of the 95% CI for those clades. For the hippocampus, in contrast, the power function that describes the mass of the structure with number of neurons does not reach significance across artiodactyls (*p* = 0.2254), and the distribution of hippocampal mass as a function of number of neurons cannot be distinguished from the distribution across afrotherians (**Figure 4F**).

### NON-NEURONAL SCALING RULES

**FIGURE 5:**
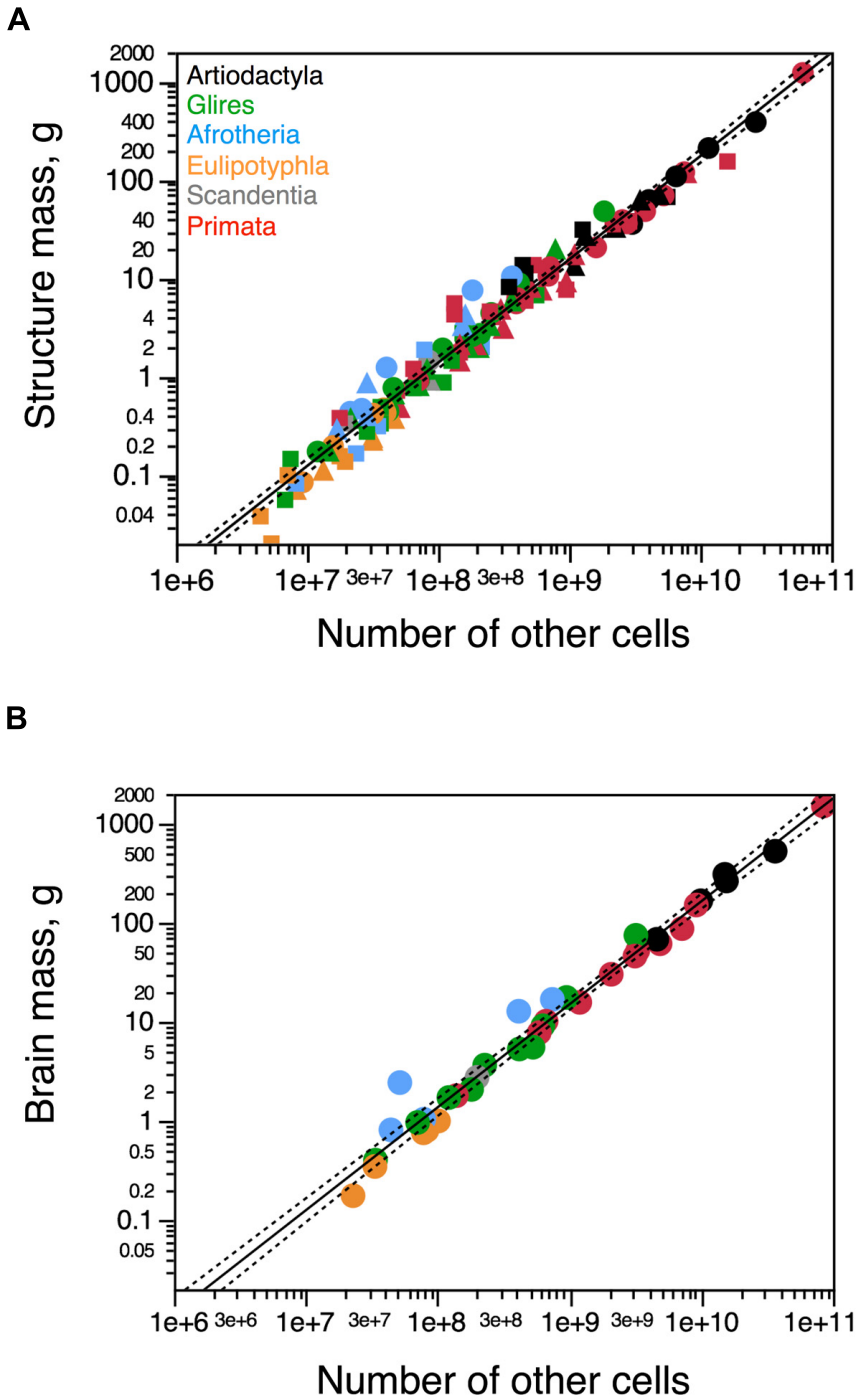
**Artiodactyls fit the non-neuronal scaling rules for all brain structures and mammalian clades.** Plots show the relationship between structure mass and number of non-neuronal (other) cells for **(A)** each brain structure or **(B)** the brain as a whole. Circles, cerebral cortex **(A)** or whole brain **(B)**; squares, cerebellum; triangles, rest of brain. Functions plotted apply to the ensemble of species, including artiodactyls. Exponents are 1.035 ± 0.017 **(A)** and 1.020 ± 0.026 **(B)**. Artiodactyl species in black, primates in red, glires in green, afrotherians in blue, insectivores in orange, scandentia in gray. Data from Herculano-Houzel et al. (2006, 2007, 2011), Azevedo et al. (2009), Sarko et al. (2009), Gabi et al. (2010), and Neves et al. (2014).

While the mass of each brain structure varies as a different power function of its number of neurons across artiodactyl species, the relationship between mass of each brain structure (cerebral cortex, cerebellum, and rest of brain) and number of other (non-neuronal) cells can be described as a single power function of exponent 0.956 ± 0.045 (*p* < 0.0001). Besides the overlap across brain structures, this distribution also overlaps with that across afrotherians, glires, eulipotyphlans as well as primates (joint exponent of 1.034 ± 0.020, *p*< 0.0001). The addition of artiodactyls to the analysis does not alter the exponent significantly (1.035 ± 0.017, *p* < 0.0001; **Figure 5A**). As a result, whole brain mass varies as a similar functions of numbers of other cells across artiodactyls (exponent, 1.104 ± 0.216, *p* = 0.0146), the ensemble of glires, primates, afrotherians, and eulipotyphlans (exponent, 1.040 ± 0.032, *p* < 0.0001), and all clades together (exponent, 1.020 ± 0.026, *p* < 0.0001; **Figure 5B**). Thus, brains of a similar size are composed of similar numbers of non-neuronal cells across different mammalian clades, from afrotherians to artiodactyls.

### CELL DENSITIES

**FIGURE 6:**
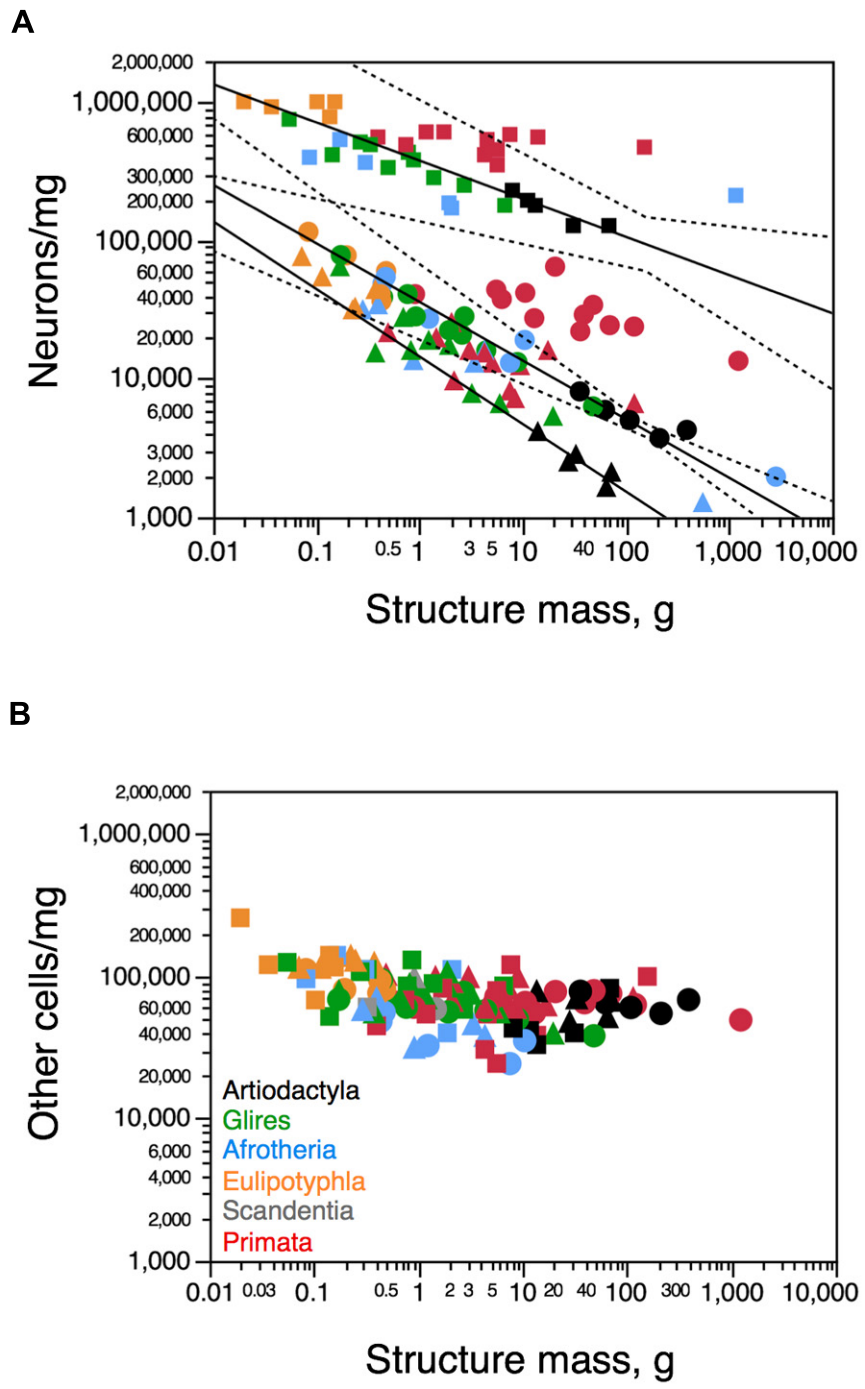
**Cellular density variation across structures and species. (A)** Average neuronal density (in number of neurons per mg of tissue) in each structure and species. **(B)** Average density of other cells (in number of other, non-neuronal cells per mg of tissue) in each structure and each species. Both graphs are plotted on a similar Y-axis for comparison. For clarity, only the significant power functions for Artiodactyla are plotted (cerebral cortex with hippocampus, exponent -0.424 ± 0.028; cerebellum, exponent -0.276 ± 0.063; rest of brain, -0.488 ± 0.113). No brain structure in any mammalian order examined here exhibits a significant correlation between other cell density and structure mass. Notice in **(A)** that the function for neuronal densities in artiodactyl cerebral cortex (minus the giraffe; circles) includes glires, afrotherians and eulipotyphlans, while the function for artiodactyl cerebellum (with the giraffe; squares) includes all other non-primate mammalian clades. Artiodactyl species in black, primates in red, glires in green, afrotherians in blue, insectivores in orange, scandentia in gray. Data from Herculano-Houzel et al. (2006, 2007, 2011), Azevedo et al. (2009), Sarko et al. (2009), Gabi et al. (2010), and Neves et al. (2014).

In artiodactyls, as in other groups of mammals, neuronal density varies considerably more than other cell density across structures (**Figures 6A,B**). Neuronal density in the cerebral cortex (gray + white matter) varies between 3,741 neurons/mg in the greater kudu to 8,118 neurons/mg in the pig; in the cerebellum, it ranges between 127,218 neurons/mg in the greater kudu to 228,632 neurons/mg in the pig; and in the rest of brain, from 1,642 neurons/mg in the greater kudu to 4,238 neurons/mg in the pig (**Table 1**). Neuronal densities in the cerebral cortex and cerebellum are lower in artiodactyl than in primates for a similar structure mass (**Figure 6A**). In contrast, other cell densities vary across all structures only between 33,123 other cells/mg in the blesbok cerebellum and 81,505 other cells/mg in the giraffe cerebellum, and overlap between artiodactyl and primate structures of similar mass (**Figure 6B**). There is no systematic correlation between variations in other cell density and in structure mass across artiodactyls species (Cx, *p* = 0.5046; Hp, *p* = 0.6000; Cb, *p* = 0.8729; RoB, *p* = 0.6238; all structures: *p* = 0.2810), as found in other clades (**Figure 6B**).

Consistently with the faster increase in structure mass than in number of neurons in the artiodactyl cerebral cortex in a manner that is similar across artiodactyls, afrotherians, glires, and eulipotyphlans, but not primates, neuronal density in the artiodactyl cerebral cortex (minus the giraffe) decreases with increasing cortical mass, as a power function of exponent -0.424 ± 0.028 (*p* = 0.0044; **Figure 6A**, circles). The 95% confidence interval for this function includes afrotherians, glires and eulipotyphlans, but excludes primates (**Figure 6A**), indicating that the scaling of neuronal densities in the cerebral cortex is shared among the former clades, but is distinct in primates. Similarly, in the artiodactyl rest of brain, neuronal density also decreases significantly as a power function of increasing structure mass (exponent, -0.488 ± 0.113, *p* = 0.0227), in a manner that cannot be distinguished from the trend in the ensemble of other clades (**Figure 6A**, triangles). Neuronal density in the artiodactyl cerebellum (including the giraffe) scales in a manner that includes most afrotherians and glires, but excludes primates, varying as a power function of cerebellar mass with exponent -0.276 ± 0.063 (*p* = 0.0222; **Figure 6A**, squares).

**FIGURE 7:**
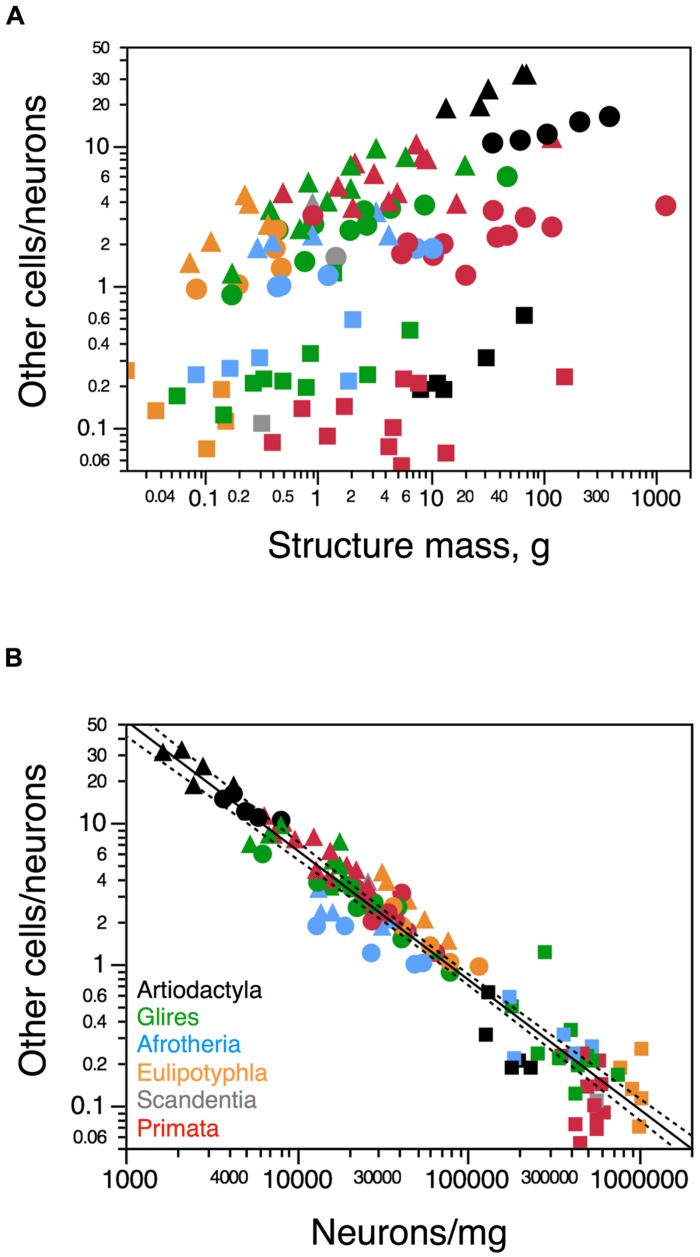
**Uniform variation in the O/N ratio with neuronal density, but not structure mass. (A)** Ratio between numbers of other (non-neuronal) and neuronal cells, O/N, varies in no uniform fashion across structures (cerebral cortex, circles; cerebellum, squares; rest of brain, triangles) and species (each symbol) as a function of the mass of each structure. **(B)** In contrast, the O/N ratio varies uniformly across brain structures and species as a function of average neuronal density in the structure. The power function plotted in b applies to the non-artiodactyl species in the sample, and has and exponent of -0.917 ± 0.028. Artiodactyl species in black, primates in red, glires in green, afrotherians in blue, insectivores in orange, scandentia in gray. Data from Herculano-Houzel et al. (2006, 2007, 2011), Azevedo et al. (2009), Sarko et al. (2009), Gabi et al. (2010) and Neves et al. (2014).

The ratio between the numbers of other cells and neurons in each structure (the O/N ratio), which approximates the glia/neuron ratio, varies between 0.184 (in the blesbok cerebellum) and 32.333 (in the giraffe rest of brain) across structures and species in these artiodactyls (**Figure 7**). The O/N ratio in the artiodactyl cerebral cortex exceeds 10 when both gray and white matter are included, and within the cortical gray matter alone it varies between 7.2 and 8.5 across species (**Table 1**). The O/N ratio varies widely across structures and species as a function of structure mass, with no single relationship evident (**Figure 7A**). In contrast, the O/N ratio varies as a common power function of neuronal density across all artiodactyl structures with an exponent of -1.082 ± 0.035 (*p* < 0.0001), in a distribution that overlaps with the variation of O/N as a function of neuronal density across non-artiodactyl species (exponent, -0.917 ± 0.028, *p* < 0.0001; **Figure 7B**). The addition of artiodactyl structures to the distribution does not change the exponent significantly (-0.935 ± 0.024, *p* < 0.0001).

### GYRIFICATION

**FIGURE 8:**
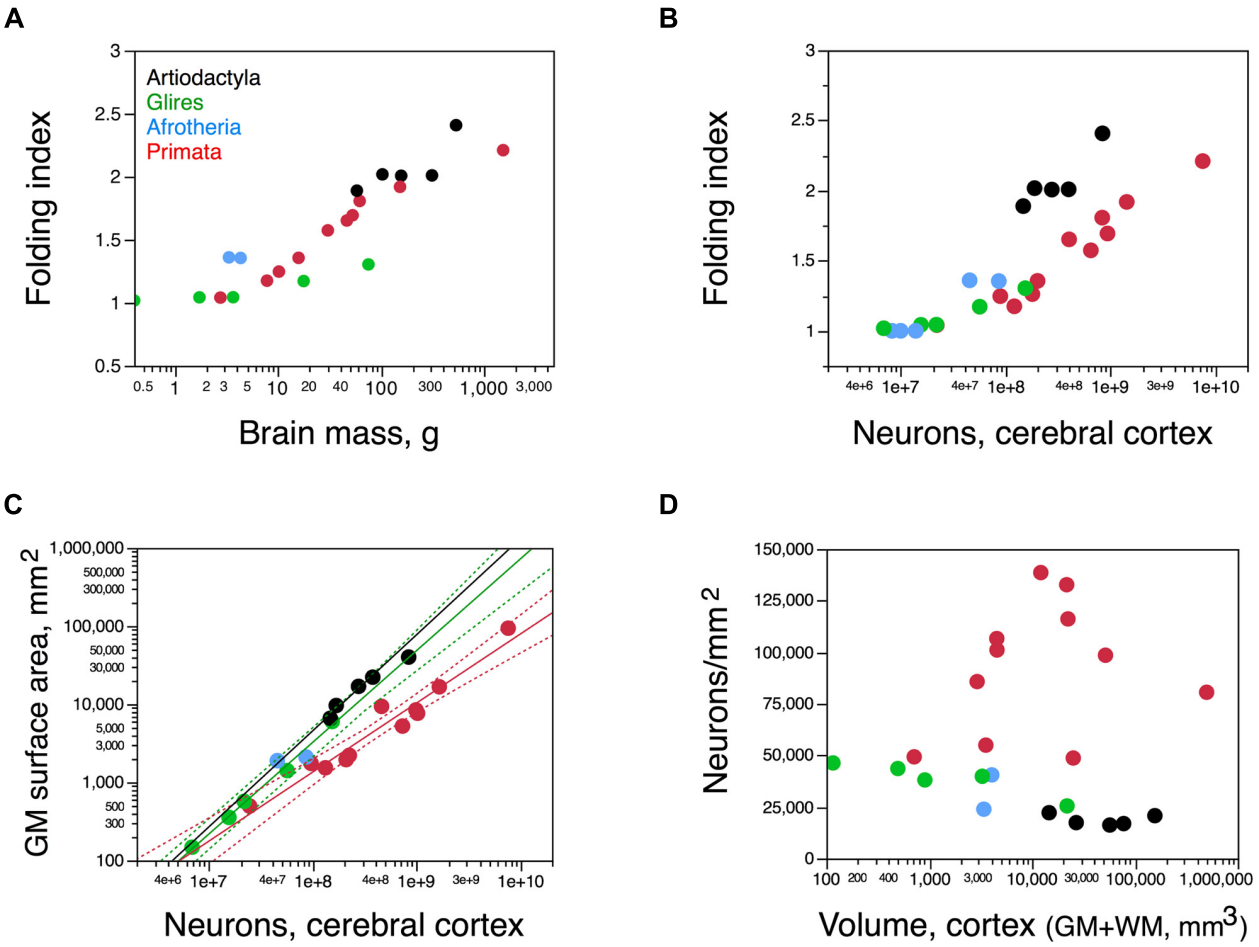
**The artiodactyl cortex is more folded than that of rodents and primates. (A)** Folding index (FI) of the cerebral cortex (ratio between the total surface area and the exposed surface area of the cortex) plotted as a function of total brain mass. **(B)** FI of the cerebral cortex plotted as a function of the number of cortical neurons in each species. Notice that for similar numbers of cortical neurons, the cortex of artiodactyls is more folded that the cortex of both primates and rodents. **(C)** Variation in total surface area of the cerebral cortical gray matter, in mm^2^, plotted as a function of the total number of neurons in the cortical gray matter. Power functions for artiodactyls (minus the giraffe), rodents, and primates are plotted and have exponents 1.230 ± 0.169, 1.174 ± 0.051, and 0.884 ± 0.071, respectively. **(D)** Variation in average number of neurons per mm^2^ of cortical surface area plotted as a function of the total volume of the cerebral cortex (gray and white matter combined). Artiodactyls in black, afrotherians in blue, glires in green, primates in red. Data from Azevedo et al. (2009), Herculano-Houzel et al. (2010), Ribeiro et al. (2013), Ventura-Antunes et al. (2013), and Neves et al. (2014).

We next examined how cortical folding and the distribution of neurons along the cortical surface compare across artiodactyls and primates species of a similar range of brain sizes (**Table 2**). We find that the cerebral cortex of artiodactyls appears to be as folded as primate cortices of a similar brain mass (**Figure 8A**), and with a distribution of folding along the anteroposterior axis of artiodactyl cortices that is similar to that observed in primates (Zilles et al., 1988; see Figure 14A). However, we find that the cerebral cortex of artiodactyls is much more folded than primate or rodent cortices of similar numbers of neurons (**Figure 8B**). For instance, while the kudu and the pig-tailed macaque monkey both have around 400 million neurons in a single cortical hemisphere, the former has a larger FI of 2.009, compared to 1.652 in the macaque (**Figure 8B**). This larger degree of folding is related to the spreading of similar numbers of neurons over a much larger cortical surface area in artiodactyls than in primates (**Figure 8C**): for instance, the ca. 400 million neurons are spread over 22,203 mm^2^ in the kudu, but over only 9,381 mm^2^ in the pig-tailed monkey.

**Table 2:** Cortical areas, volumes and folding index for artiodactyls.

Species	A_GM_, mm^2^	V_GM_, mm^3^	V_WM_, mm^3^	FI
*Sus scrofa domesticus*	6,594	8,807	5,834	1.889
*Antidorcas marsupialis*	9,623	16,063	10,809	2.018
*Damaliscus dorcas phillipsi*	16,915	36,692	20,330	2.007
*Tragelaphus strepsiceros*	22,203	52,041	25,691	2.010
*Giraffa camelopardalis*	40,128	96,048	60,309	2.408

*A_GM_, pial surface of the cortical grey matter; V_GM_, volume of the cortical grey matter; V_WM_, volume of the subcortical white matter; FI, folding index. All values refer to the results for one single hemisphere.*

As a consequence, the average number of neurons underneath 1 mm^2^ of cortical surface, N/A, is 2–6 times smaller in artiodactyls (18,706 ± 1,176 neurons/mm^2^) than in primates (between 40,000 and 120,000 neurons/mm^2^; **Figure 8D**). The ratio N/A in artiodactyls does not scale with brain size, number of cortical neurons, cortical volume or surface area (Spearman correlation, *p* = 0.6238; **Figure 8D**).

**FIGURE 9:**
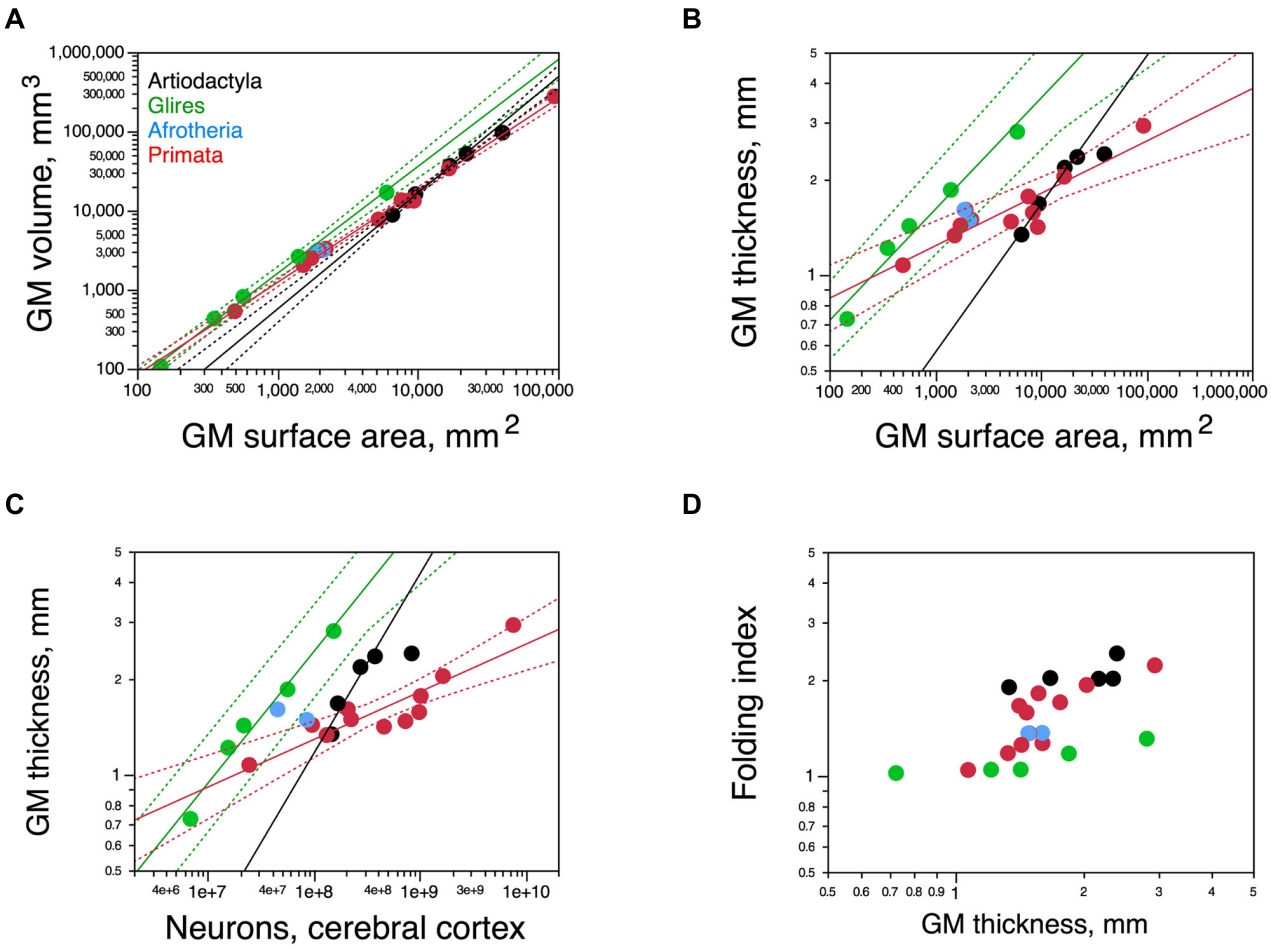
**Scaling of the cortical gray matter. (A)** Variation in the volume of the cortical gray matter as a function of the total surface area of the gray matter. Power functions for artiodactyls (minus the giraffe), rodents, and primates are plotted and have exponents 1.466 ± 0.036, 1.350 ± 0.037, and 1.165 ± 0.026, respectively. Notice that the artiodactyl cortex has a larger surface area than rodents for a similar gray matter volume. **(B)** Variation in the thickness of the cortical gray matter as a function of the total surface area of the gray matter. Power functions for artiodactyls (minus the giraffe), rodents, and primates are plotted and have exponents 0.466 ± 0.036, 0.350 ± 0.037, and 0.165 ± 0.026, respectively. For a similar surface area, the artiodactyl cortex is thinner than the rodent cortex. **(C)** Variation in the thickness of the cortical gray matter as a function of the total number of cortical neurons. Power functions for artiodactyls (minus the giraffe), rodents, and primates are plotted and have exponents 0.562 ± 0.118, 0.413 ± 0.040, and 0.149 ± 0.024, respectively. Again, for a similar number of cortical neurons, the artiodactyl cortex is thinner than the rodent cortex. (**D)** FI of the cerebral cortex plotted as a function of the average thickness of the cortical gray matter. Notice that for similar cortical thicknesses, both the artiodactyl and the primate cortex are more folded than the rodent cortex. Artiodactyls in black, afrotherians in blue, glires in green, primates in red. Data from Azevedo et al. (2009), Herculano-Houzel et al. (2010), Ribeiro et al. (2013), Ventura-Antunes et al. (2013), and Neves et al. (2014).

Cortical surface area increases with numbers of neurons raised to exponents of 1.230 ± 0.169 across artiodactyls (minus the giraffe) and 1.174 ± 0.051 across rodents, but only 0.884 ± 0.071 across primates (**Figure 8C**). Although the relationship between cortical surface area and numbers of neurons seems overlapping in a log-log scale between artiodactyls and glires (**Figure 8C**), the strikingly different folding of the cortical surface suggests a different distribution of the cortical volume and its neurons along the surface. Indeed, there are three distinct patterns of surface distribution of the cortical volume in rodents, primates, and artiodactyls, visible in the relationship between cortical gray matter volume and gray matter surface area (**Figure 9A**). Gray matter volume increases with gray matter surface area raised to the power of 1.466 ± 0.036 in artiodactyls (minus the giraffe; *p* = 0.0006), 1.165 ± 0.026 in primates (*p* ≤ 0.0001), and 1.350 ± 0.037 in rodents (*p* < 0.0001; **Figure 9A**). The different surface spreading of the cortical volume is more clearly apparent in the relationships between cortical gray matter thickness and surface area (**Figure 9B**) and between cortical thickness and number of cortical neurons (**Figure 9C**). Gray matter thickness increases with cortical surface area raised to the power of 0.466 ± 0.036 in artiodactyls (minus the giraffe; *p* = 0.0059), 0.350 ± 0.037 in rodents (*p* = 0.0025), and 0.165 ± 0.026 in primates (*p* = 0.0001). Gray matter thickness increases with numbers of cortical neurons raised to the power of 0.562 ± 0.118 in artiodactyls (minus the giraffe; *p* = 0.0413), 0.413 ± 0.040 in rodents (*p* = 0.0019), and 0.149 ± 0.024 in primates (*p* = 0.0001). These analyses show that the cortical volume is spread more thinly in artiodactyls than in rodents, with a thinner cortex in artiodactyls for a similar number of neurons (**Figure 9C**) or surface area (**Figure 9B**). Although artiodactyls and primates have some overlap between cortical thickness and surface area (**Figure 9B**), similar numbers of cortical neurons are spread more thinly in primate than in artiodactyls (**Figure 9C**). Yet, the thicker artiodactyl cortices (for a similar number of neurons; **Figure 9C**) are more folded than primate cortices of either similar numbers of neurons (**Figure 8B**) or similar thickness (this is the case for the pig, blesbok, and giraffe; **Figure 9D**), which indicates that the differential thickness of the cerebral cortex is not determinant of the degree of cortical folding.

### DISTRIBUTION OF NEURONS WITHIN THE CEREBRAL CORTEX

**FIGURE 10:**
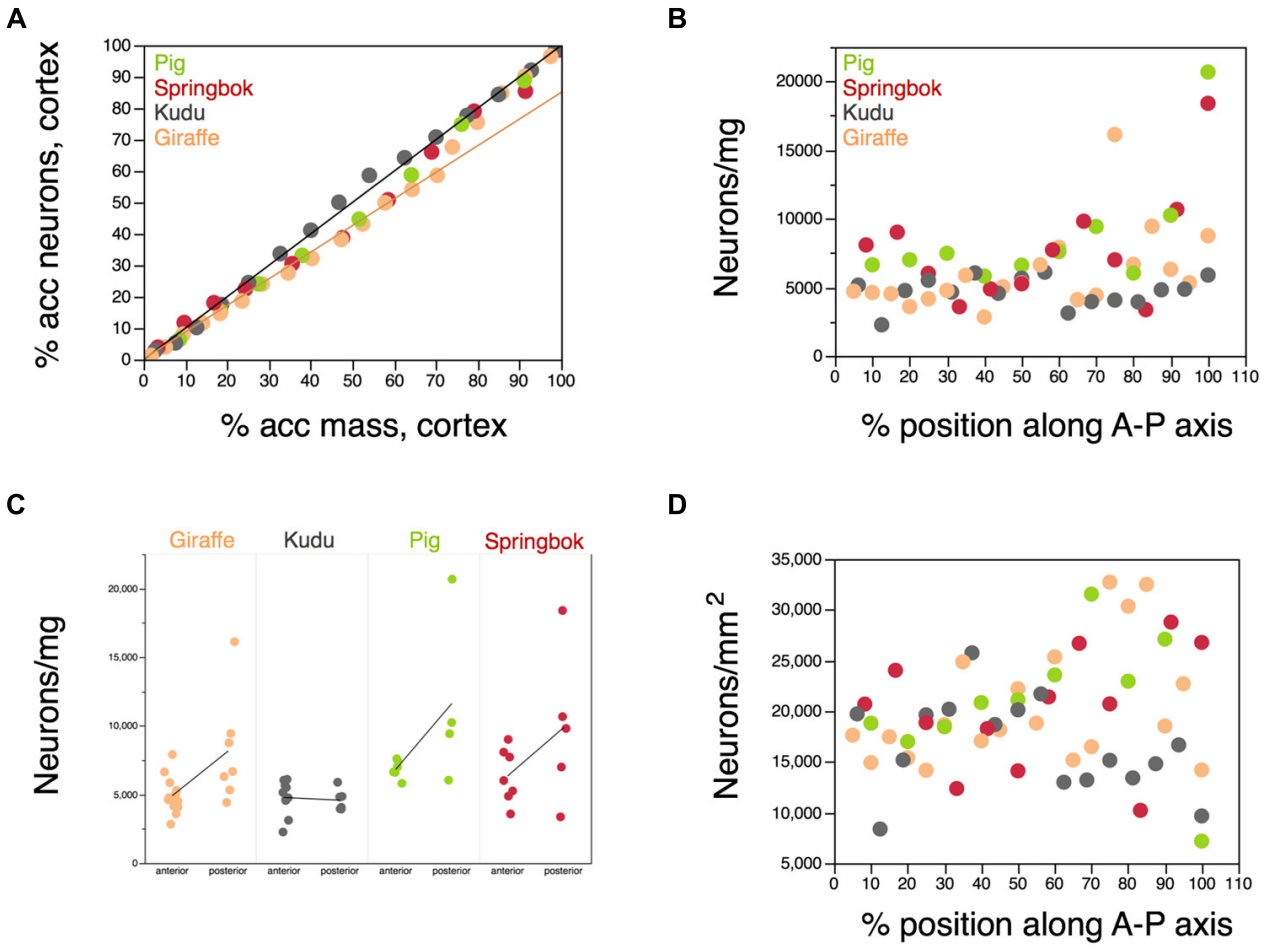
**Distribution of neurons along the anteroposterior axis of the cerebral cortex.** Each artiodactyl species is represented in a different color. **(A)** Cumulative percentage of neurons along the anteroposterior axis of the cerebral cortex plotted as a function of the cumulative mass of the gray matter of the cerebral cortex along the same axis. Anterior is to the left. Each point represents the values for one 6 mm block of cortex along the axis. The plotted line indicates the linear relationship expected for a uniform distribution of neurons in the cortical mass along the anteroposterior axis. While the kudu fits the uniform distribution, the springbok, pig, and giraffe deviate in such a manner that the cumulative distribution of neurons along the cortical mass is better described by a different function (plotted) in the anterior 2/3 of the cortical mass. **(B)** Variation in neuronal density in each 6 mm block of cerebral cortex along the anteroposterior (A-P) axis. Anterior is to the left. While there is little variation in neuronal density along the cortex of the kudu, densities vary by 4–5× among cortical blocks in the pig, springbok and giraffe. **(C)** Difference in neuronal densities found in the anterior 2/3 and in the posterior 1/3 of the cerebral cortex in each species. Averages in each group are shown for each species. The largest neuronal densities are found in the posterior 1/3 of the cortex in the giraffe, pig, and springbok. **(D)** Variation in the surface density of neurons in the cortex (that is, neurons/mm^2^) in each 6 mm block of cerebral cortex along the anteroposterior axis. Despite the large variation along the cortex of each species, there is no systematic difference in surface density of neurons along the A-P axis.

If neurons were distributed evenly across the cortical mass in the five artiodactyl species, then the accumulated number of neurons along the anteroposterior axis should vary linearly with the accumulated mass of the cortical gray matter along the same axis and this distribution should be similar across species. In contrast, while in the kudu the distribution of cortical neurons is mirrored in the distribution of cortical mass (**Figure 10A**, gray points), in the pig, in the springbok, and in the giraffe, there is a slower accumulation of neurons along the anterior two thirds of the cortex, and a faster accumulation of neurons along the posterior third of the cortex. This suggests that, as was observed in the human cortex (Ribeiro et al., 2013), the cerebral cortex of artiodactyls (although not of the kudu) has a larger density of neurons in the posterior regions. Indeed, the neuronal density in the cortical gray matter increases significantly from the anterior to the posterior pole across the four species combined (Spearman correlation, ρ = 0.3121, *p* = 0.0171; **Figure 10B**). Within each species, however, the correlation is only significant for the giraffe (ρ = 0.6211, *p* = 0.0035), and it is only in the giraffe that a one-way ANOVA shows a significant difference in neuronal density between the anterior two thirds and the posterior third of the cortex (giraffe, *p* = 0.0136; pig, *p* = 0.0969; springbok, *p* = 0.1516; kudu, *p* = 0.7337; **Figure 10C**). Still, in the pig and springbok, despite the non-significant correlation between neuronal density and position along the anteroposterior axis (pig, *p* = 0.1175; springbok, *p* = 0.3911), the posteriormost block of cerebral cortex is the portion that exhibits the largest neuronal density of all, with values about 4× larger than in the remainder of the cortex (**Figure 10B**). In the giraffe, the block with the largest neuronal density is situated 70% along the anteroposterior axis (**Figure 10B**).

In contrast to the trend toward larger neuronal densities in the posterior cortex, there is no correlation between the distribution of neurons per cortical surface area (neurons/mm^2^) and position along the anteroposterior axis (ρ = 0.6211, *p* = 0.0035; **Figure 10D**). While this does not imply that neurons are distributed evenly across the cortical surface, the lack of correlation suggests that there is no systematic variation in the distribution of neurons under the cortical surface along the anteroposterior axis.

**FIGURE 11:**
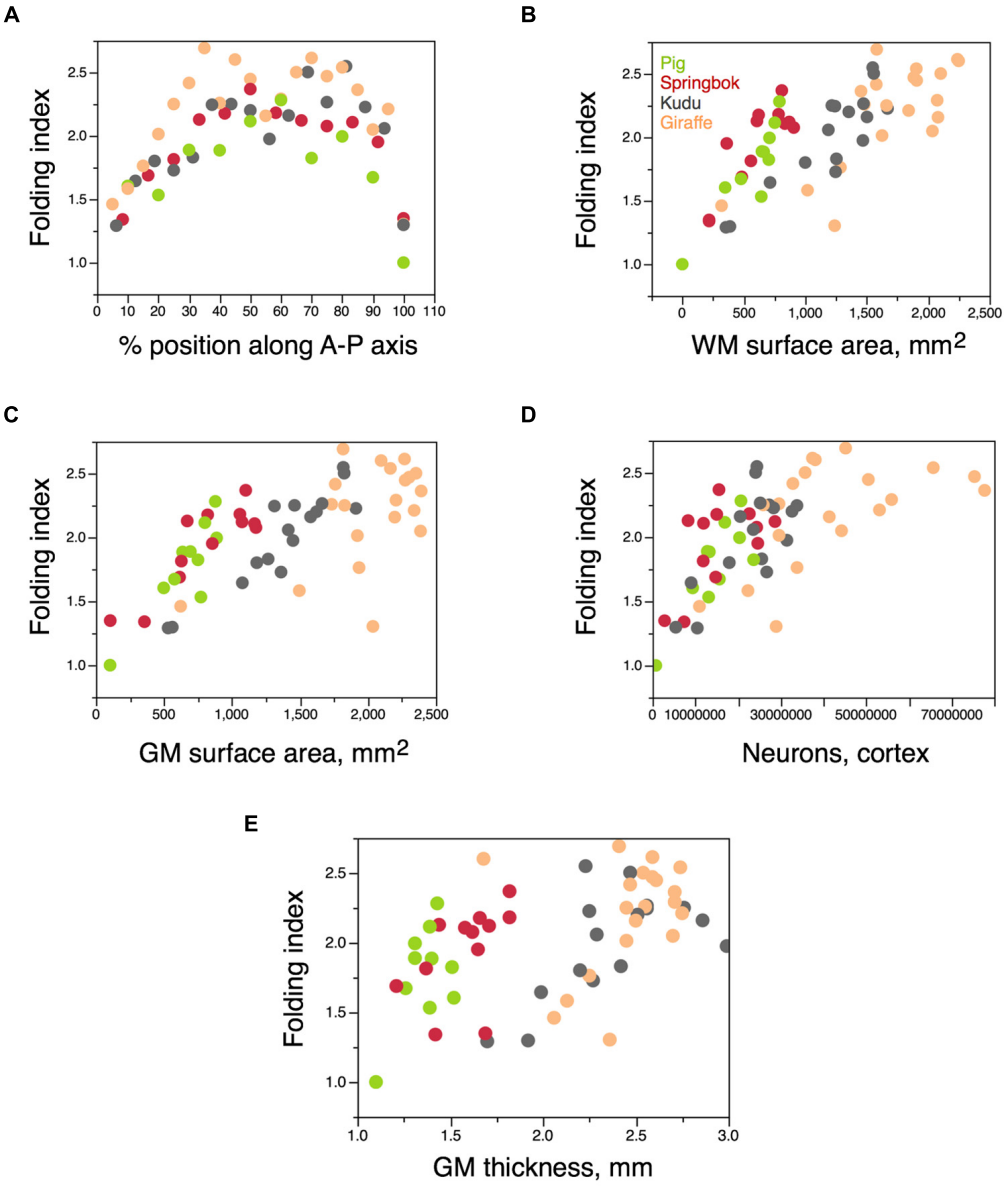
** Variation in FI within the cerebral cortex.** Each artiodactyl species is represented in a different color. **(A)** Variation in FI of the cortex along the anteroposterior axis of the cerebral cortex. Anterior is to the left. Each point represents the values for one 6 mm block of cortex along the axis. The smallest folding indices in the poles are a trivial consequence of the shape of the cortex in relation to the axis of measurement (that is, of the surface becoming orthogonal to the axis at the poles), but the overall shape of the distribution indicates a larger folding of the cortex in the intermediate positions along the A-P axis in artiodactyls, as in primates (Zilles et al., 1988). **(B–E)** Variation in FI amongst blocks of cerebral cortex along the A-P axis for each species as a function of the surface area of the white matter in the block **(B)**, surface area of the gray matter in the block **(C)**, number of cortical neurons in the block **(D)**, or average thickness of the gray matter in the block **(E)**.

In these four artiodactyl species, the degree of folding of the cortical surface varies about 2× along the anteroposterior axis (**Figure 11A**), in the same manner found in the human cerebral cortex, with less folding in the poles than in the center (Zilles et al., 1988; Ribeiro et al., 2013). Across all four species, variations in the degree of folding along the anteroposterior axis are correlated best with variations in the surface area of the white–gray matter interface (ρ = 0.7459, *p* < 0.0001; **Figure 11B**), but also with variations in the surface area of the gray matter (ρ = 0.6822, *p* < 0.0001; **Figure 11C**), in numbers of cortical neurons (ρ = 0.6721, *p* < 0.0001; **Figure 11D**) and in cortical thickness (ρ = 0.5361, *p* < 0.0001; **Figure 11E**). Notice that the correlation with cortical thickness is positive, that is, regions where the cortical gray matter is thicker tend to be more folded. Similarly, within each species separately, the best predictor of variations in FI along the anteroposterior axis is the surface area of the white–gray matter interface (all species, ρ > 0.6, *p* < 0.02; **Figure 11B**); gray matter surface area is not correlated with FI in the giraffe (*p* = 0.1906), numbers of cortical neurons are not correlated with FI in the springbok (*p* = 0.1121), and cortical thickness is not correlated with FI in the giraffe (*p* = 0.1844) and in the pig (*p* = 0.5772). Thus, variations in cortical folding along the cortical surface appear to be best predicted by variations in the extent of the white–gray matter surface – although the relationship is not a single one for the four species of artiodactyls (**Figure 11B**).

### PREDICTIONS FOR CETACEANS

Artiodactyls are members of the order Cetartiodactyla, which includes the closely related cetaceans, large animals with large brains. So far, we have found that neuronal scaling rules apply to all species within a given order (primates, rodents) or superorder (afrotherians), and even across orders, as in the case of the cerebral cortex of rodents, lagomorphs, afrotherians, and artiodactyls (Herculano-Houzel et al., 2014). Because the total mass of the artiodactyl cerebral cortex conforms to the neuronal scaling rules that apply to the ensemble of artiodactyls (minus the giraffe), glires, and afrotherians, and given that cetaceans and artiodactyls belong to the same mammalian order, the rules that apply to the ensemble of glires, afrotherians, and artiodactyls can be used to predict the neuronal composition of the cetacean cerebral cortex. The prediction is given by the equation N_CXT_ = e^17.327±0.045^. M_CXT_^0.590±0.018^, where N_CXT_ is the total number of neurons in the cerebral cortex and M_CXT_ it the total mass of the cerebral cortex (gray + white matter, in g). This equation is obtained for the relationship between cortical mass and number of neurons across glires, afrotherians, and artiodactyls (minus the giraffe) written to have number of cortical neurons as a dependent variable and cortical mass as the independent variable. Using the cortical volumes given in cm^3^ by Hofman (1985), which can be approximated to cortical mass in grams (gray + white matter combined), we predict the cerebral cortex of *Phocoena phocoena*, *Tursiops truncatus*, *Grampus griseus*, and *Globicephala macrorhyncha*, at 340, 815, 1,127, and 2,045 cm^3^, to be composed of 1.04, 1.75, 2.11, and 3.01 billion neurons, respectively.

## DISCUSSION

Here we determined the numbers of neuronal and non-neuronal cells that compose the brains of five artiodactyl species. We find that the artiodactyl cerebral cortex and cerebellum conform to the neuronal scaling rules that apply to afrotherians, glires, and eulipotyphlans, which we have suggested also apply to the last common ancestor of modern eutherians (Herculano-Houzel et al., 2014; Neves et al., 2014). Although the volume of the cerebral cortex is a shared function of its number of neurons across artiodactyls, afrotherians, rodents, and eulipotyphlans, the surface distribution of this volume differs across clades, resulting in widely different numbers of neurons per unit of surface area across the species, and in cortices that fold to different extents in different species, even when composed of similar numbers of neurons. These dissimilarities underline one common principle in brain evolution that becomes more and more clear: that there is no single common principle regarding how neurons build the brain, such that all mammalian brains are not the same (Herculano-Houzel, 2011), although patterns are beginning to emerge that illuminate the ways in which mammalian brains have evolved (Herculano-Houzel et al., 2014).

We will next discuss how the simple determination of the cellular composition of the brain of five artiodactyl species, in comparison to a total of 31 other mammalian species, illuminates several of the most basic aspects of brain organization, development, and evolution.

### NEURONAL SCALING RULES

Upon realizing that even closely related clades such as rodents and primates had brain structures formed according to strikingly different neuronal scaling rules (Herculano-Houzel et al., 2006, 2007), we had initially supposed that each mammalian order might have its own characteristic set of neuronal scaling rules dictating how the size of each brain structure scales with number of neurons in the structure. While we then found eulipotyphlans to share neuronal scaling rules for the cerebral cortex, but not for the cerebellum, with rodents and lagomorphs (that is, glires; Sarko et al., 2009; Herculano-Houzel et al., 2011), we recently found that the afrotherian neuronal scaling rules for both the cerebral cortex and the cerebellum are also shared with glires (Neves et al., 2014). Given the position of Afrotheria closer to the base of the Eutheria evolutionary tree, the sharing of neuronal scaling rules for the cerebral cortex by afrotherians, glies, and eulipotyphlans raised the possibility that those were the ancestral rules that applied to the formation of the cerebral cortex of mammals. The present finding that the neuronal scaling rules for the cerebral cortex and cerebellum of afrotherians and glires are also shared by artiodactyls strengthens this possibility and suggests that primates and eulipotyphlans diverged from the ancestral rules, which however still apply to Artiodactyla (Herculano-Houzel et al., 2014).

The existence of ancestral neuronal scaling rules that remained conserved in the evolution of some clades is further supported by the finding that the rules that apply to the rest of the brain (brainstem plus diencephalon and basal ganglia), which are evolutionarily older structures than the cerebral cortex, are shared by afrotherians, glires, eulipotyphlans, and even primates – although possibly not by artiodactyls, a more recent group than the others.

Unfortunately, the giraffe brain specimen that we had available for analysis was not from a fully grown adult, but only a juvenile, of about half the expected adult body mass and subadult brain mass (470 and 528 g compared to 1,200 and 700g, respectively; Boddy et al., 2012). Although we do not know how artiodactyl development compares to rodents and primates, we presume that the final stages of development that expand brain mass, with extension of arbors, resulting in increased neuronal cell mass, and with addition of large numbers of non-neuronal cells, were not yet completed, although the mechanisms that determine final numbers of neurons were possibly already completed (Bandeira et al., 2009), such that the average total number of neurons in the giraffe brain is possibly well represented by the numbers found here. Such an incomplete developmental brain expansion would provide a simple explanation for our findings of structure masses that are well below the expected values for the numbers of neurons in the structures in comparison to the other artiodactyls in the sample. It was based on this rationale that we excluded the giraffe data from the analyses, although they were presented for comparison.

### NON-NEURONAL SCALING RULES

We show that artiodactyls share non-neuronal scaling rules not only with afrotherians and glires, but also with eulipotyphlans and even with primates, and across all brain structures. “Non-neuronal cells” are not only glial cells (of all different types) but also endothelial cells and pericytes. However, given that the vascular volume has been measured at less than 3% of the cerebral cortex of the human brain (Lauwers et al., 2008) as well as in rodents and other primates (reviewed in Tsai et al., 2009), we presume that glial cells are the vast majority of non-neuronal cells. Thus, the shared relationship between the mass of brain structures and their numbers of non-neuronal cells indicates that glial cells are added to different brain structures in different species following similar rules and mechanisms that have been conserved in evolution. This is illustrated by the finding that primate and artiodactyl brains of similar sizes are composed of strikingly different numbers of neurons, but similar numbers of non-neuronal (mostly glial) cells. For example, the pig brain, at 68.2 g, has 2.2 billion neurons, almost half than the slightly smaller bonnet monkey brain, at 62 g, which has 3.8 billion neurons (Gabi et al., 2010), but both have fairly similar numbers of other cells: 4.6 billion in the pig, 4.9 billion in the bonnet monkey. Such evolutionary conservation of glial scaling rules, especially in the face of diversity in the neuronal scaling rules, suggests that physiological constraints are in place that limit diversification in how glial cells, of all glial subtypes, are added to the neuronal parenchyma in development (Herculano-Houzel, 2014; Mota and Herculano-Houzel, 2014).

One consequence of the evolutionarily conserved, shared glial scaling rules is the uniform increase in the O/N ratio, and thus in the glia/neuron ratio, with decreasing neuronal density. We have shown that the glia/neuron ratio increases as a consequence of increasing average neuronal cell mass following the same rules across brain structures and species (Mota and Herculano-Houzel, 2014). In the present artiodactyls, we find glia/neuron ratios that exceed 7–8 within the cortical gray matter, 10 in the cerebral cortex as a whole, and 20 or even 30 in the brainstem. Notice that these artiodactyl cortices reach at most 1/3 of the mass of the human cerebral cortex, and yet have glia/neuron ratios in the cortical gray matter that are as much as 5× larger than in the human gray matter (Sherwood et al., 2006; Azevedo et al., 2009). This difference illustrates that the glia/neuron ratio is not a function of brain mass or cortical mass, but rather of neuronal density, which may or may not vary with cortical mass, as we have pointed out before (Herculano-Houzel, 2011, 2014).

### COMPARISON WITH PRIMATES

One result of the scaling of the artiodactyl cerebral cortex conforming to the neuronal scaling rules that relate structure mass to numbers of neurons in afrotherians and glires, but not primates, is that the cerebral cortex is much larger in artiodactyls than in primates with similar numbers of neurons. For instance, the springbok and the owl monkey have cerebral cortices with around 400 million neurons (springbok, 375 million; owl monkey, 442 million), but the springbok cortex, at 62.8 g, is nearly 6× larger than the owl monkey cortex, which weighs only 10.6 g (Herculano-Houzel et al., 2007). Similarly, the kudu and the pig-tailed monkey both contain ca. 800 million neurons in the cerebral cortex, but the structure weighs 212 g in the kudu, in contrast to only 36.2 g in the macaque (Gabi et al., 2010); and the giraffe has as many cortical neurons as a rhesus monkey (1.7 billion), but in a structure that weighs 390 g in a juvenile, versus only 70 g in an adult rhesus monkey (Herculano-Houzel et al., 2007). The gorilla, the primate species with a comparable cortical mass to the giraffe, has, in contrast, an estimated 8.9 billion cortical neurons (Herculano-Houzel and Kaas, 2011), or 5× more neurons in the cerebral cortex than the giraffe. We speculate that these large differences in numbers of cortical neurons result in very different cognitive abilities between artiodactyl and primate species of similar cortical mass such as the giraffe and the gorilla (at ∼400 g), and the pig and the pig-tailed monkey (at ∼36 g), potentially endowing primates with more processing power, behavioral complexity, and flexibility than their artiodactyl counterparts.

Moreover, non-sensory, non-motor and thus presumably associative cortical areas in artiodactyls are limited to the very frontal pole of the cortex (Welker et al., 1976), while in great apes (and human) they amount to 14% of the cortical gray matter volume (Schoenemann et al., 2005). Thus, the distribution of similar numbers of neurons between sensorimotor and associative areas is also likely to contribute to the different cognitive abilities of artiodactyls and primates with similar numbers of cortical neurons. When compounded with the larger average size of neurons in artiodactyls than in primates for a similar number of cortical neurons, we predict that the gorilla not only has 5× more cortical neurons overall than the giraffe, for a similar cortical mass, but it has even more than 5× more neurons available for associative (prefrontal) processing, rather than purely sensorimotor processing, than the giraffe. We are now examining these differences across clades in numbers of associative × sensorimotor neurons directly.

Our data show that the larger cortical volume in artiodactyls than in primates for a similar number of neurons is not simply attributable to a disproportionately larger white matter. While a similar number of cortical neurons is indeed accompanied by a nearly 10× larger volume of white matter in artiodactyls than in primates, those neurons reside in a cortical gray matter that is also nearly 10× larger in volume in artiodactyls than in primates, such that the relationship between gray and white matter volumes is overlapping across both clades. The difference is that the same volumes of gray or white matter comprise far fewer neurons in artiodactyls than in primates. Given that non-neuronal densities are comparable across the clades, it can be inferred that artiodactyl neurons, with cell parts in both gray and white matters, are about 10× larger in artiodactyls than in primates. Our results thus demonstrate that there is not a single way of building a large cerebral cortex in nature. Still, the fairly overlapping distributions of gray and white matter volumes across clades suggest the existence of common principles underlying the organization of the two portions of the cortical matter, that is, principles that link the volume of white matter to the volume of the gray matter. As we demonstrate here, however, such common principles are not related simply to sheer numbers of cortical neurons; instead, we suggest that they are related directly to the volume of the gray matter, which in turn results from variations in both number and average size of the neurons therein.

### DISTRIBUTION OF CORTICAL NEURONS WITHIN THE CORTICAL SURFACE AND IMPLICATIONS FOR CORTICAL EXPANSION IN EVOLUTION

We have previously shown that the visual cortex of mouse and man has a different neuronal composition that the rest of the cerebral cortex (Herculano-Houzel et al., 2013; Ribeiro et al., 2013), in agreement with previous findings (Rockel et al., 1980), which prompted us to perform a similar systematic analysis of the neuronal composition of the cortical volume in artiodactyls. Our current analysis, however, was hampered by the shortage of data on functional localization in the cerebral cortex of artiodactyls and by the disagreement between the few sources available regarding the position of the visual cortex. Using microelectrode recordings in the llama, Welker et al. (1976) placed its visual cortex medial to the suprasylvian fissure, along the dorsalmost cortical surface; the auditory cortex lateral to the suprasylvian fissure; and somatosensory areas rostrally in the cortex, although not extending all the way to the frontal pole. In contrast, Clarke and Whitteridge (1976), studying the sheep, placed the visual cortex in the occipitalmost areas, as found in primates, much more posteriorly than described by Welker et al. (1976).

Our findings of a trend toward larger neuronal densities in the posterior third than in the anterior two thirds of the cortex is compatible with an occipital location of a visual cortex with a distinctly high neuronal density, at least in some species, such as the pig and springbok. However, the largest neuronal densities occur in the posteriormost cortex of the pig and springbok, but at about 70% of the giraffe, while there is no section with a noticeably larger neuronal density in the kudu. While these differences may be due to a lack of resolution in our sampling, for instance because a dorsal (rather than occipital) visual cortex would have been mixed with non-visual areas in the same sections, they are compatible with a scenario in which the location of the visual cortex is not shared across artiodactyl species, being occipital in the pig and springbok (as in the sheep; Clarke and Whitteridge, 1976) but more rostral in the giraffe (and llama; Welker et al., 1976). Thus, while we show evidence of heterogeneity in the distribution of neurons along the cortical surface in three of four species of artiodactyls, with zones of much higher neuronal densities than in the remainder of the cortex, there is not enough evidence at present to assign these regions of higher neuronal density to the visual cortex.

In contrast, we do show that there is no systematic variation in the average number of neurons per surface area of cerebral cortex along the anteroposterior axis, contrary to what has been found in the cortex of human (Ribeiro et al., 2013) and non-human primates (Cahalane et al., 2012), and to what has been proposed by Barbara Finlay’s group to be the basis of the expansion of the cortical surface: a front-to-back gradient in the start of neurogenesis, which would lead to a front-to-back gradient in the surface density of neurons (Cahalane et al., 2012). If such a gradient in neurogenesis does indeed lead to a matching gradient in the surface density of neurons, then our data suggest that there is no gradient in the timing of neurogenesis in the formation of the artiodactyl cortex. Alternatively, the existence of such a gradient in the development of the artiodactyl cortex would provide evidence that a rostrocaudal gradient in the timing of neurogenesis does not necessarily lead to a gradient in the surface density of neurons along the cortex.

It has been suggested that the extension of the developmental interval required to give rise to larger total numbers of cortical neurons across species would occur through a combination of (1) an increase in the difference in timing of neurogenesis in rostral versus caudal cortex and (2) late-born supragranular neurons becoming disproportionately more numerous in the caudal than in the rostral pole relative to early born infragranular neurons (Charvet et al., 2013). Such a combination would supposedly result necessarily in steeper gradients in the surface density of neurons in those cortices with more neurons, such as in primates compared to rodents (Charvet et al., 2013). Thus, if such a mechanism in which the extension of neurogenesis necessarily leads to steep gradients in the surface density of neurons were the universal mechanism whereby cortical expansion occurs across mammalian species, then cerebral cortices with larger numbers of neurons should necessarily exhibit steeper gradients than cortices with fewer neurons. In contrast, here we find that the pig and the springbok, with numbers of cortical neurons in the range between a marmoset and an owl monkey, and the kudu, with more cortical neurons than the owl monkey, exhibit no significant variation in surface density of neurons along the rostrocaudal axis of the cerebral cortex, in contrast to the primate species with similar numbers of cortical neurons, which do exhibit sharp gradients (Charvet et al., 2013). The discrepancy indicates that, contrary to the proposition of Charvet et al. (2013), there is no single mechanism for expanding the cerebral cortex across mammals; an overall larger number of neurons may (as in primates) or may not (as in artiodactyls) occur in the presence of a rostrocaudal gradient in the surface density of neurons.

Instead, we suggest that such gradients result from variations in connectivity-related parameters that determine the extent of cortical surface formed by a given number of neurons, depending on their lateral spread, such as the fraction of gray matter neurons that are connected through the white matter and the average caliber of those fibers (Mota and Herculano-Houzel, 2012). Thus, given a different pattern of connectivity of cortical neurons through the white matter (amongst other variables, such as the average size of the neurons in the gray matter), similar numbers of cortical neurons may give rise to smaller or larger cortical surfaces in different species, with or without establishing a gradient in surface density along the rostrocaudal axis.

Finally, the increasing degree of gyrification that is found to accompany increasing cortical volume (Hofman, 1985) is usually credited to the expansion of the number of neurons in the cerebral cortex, whether homogeneously, through the lateral addition of columnar modules (Rakic, 1988) or for instance via a conical expansion of the outer subventricular zone (Lewitus et al., 2013). In contrast, here we show that the artiodactyl cerebral cortices are more folded than primate cerebral cortices of similar numbers of neurons. Thus, the degree of cortical gyrification is not necessarily proportional to the number of neurons in the cortex, and for this reason, the mechanisms driving cortical folding must be sought in events other than simply the regulation of neurogenesis. This will be the subject of a separate investigation (Mota and Herculano-Houzel, 2014).

### PREDICTIONS FOR CETACEANS

Here we examined only the artiodactyl branch of the Cetartiodactyla; determining whether the present scaling rules that apply to artiodactyls extend to the whole of Cetartiodactyla will require examining cetacean brains, which we are undertaking now. Still, given our previous findings that species in the same order (or even superorder, as is the case of Afrotheria; Neves et al., 2014) share the same neuronal scaling rules, it is plausible that the rules that we found here for artiodactyls will also apply to cetacean brains.

If this is the case, we predict that the large cerebral cortex of several cetacean species, such as the pilot whale *Globicephala macrorhyncha*, which is about twice larger than the human cerebral cortex, is composed of only around 3 billion neurons. This is at odds with a previous stereological estimate of 13 billion neurons in the cerebral cortex of the minke whale, *Balaenoptera acutorostrata* (Eriksen and Pakkenberg, 2007), at a density of about 8,000 neurons/mm^3^ of its 1,622 cm^3^ of gray matter. The same group later estimated an even larger total of 15 billion neurons in the smaller cerebral cortex of the harbor porpoise (Walloe et al., 2010). Those studies, however, may have been biased by undersampling, given that the authors sampled only 12–13 sections out of over 3,000 sections of the minke whale cerebral cortex, counting a total of only about 100 disectors per entire cortex, with a total of around 200 cells sampled from the entire cortex (Eriksen and Pakkenberg, 2007; the study on the harbor porpoise does not provide information on the sampling fraction and other parameters used). In contrast, our method samples the entire cerebral cortex, which removes any concerns of bias due to undersampling. The neuronal density that Eriksen and Pakkenberg (2007) report in the cortical gray matter of the minke whale, 8,000 neurons/mm^3^, is even higher than those densities we find in much smaller cerebral cortices of other cetartiodactyls (see **Table 1**), whereas, given the rapid decrease in neuronal density with increasing cortical mass, we would expect densities of no more than 5,000 neurons/mm^3^, and probably fewer, in cetacean cortices. Thus, there are two possible scenarios: that Eriksen and Pakkenberg’s (2007) estimate is inflated by undersampling, which would leave open the possibility that cetaceans do fit the rules reported here for other Cetartiodactyls; or that our estimates are underestimates, and cetaceans have larger neuronal densities and total numbers of neurons than can be predicted from the scaling rules reported here. However, given that we sample the entire cerebral cortex using a method that has been shown to be as accurate as stereology (Bahney and von Bartheld, 2014), while they use stereology to sample only a handful of sections out of over 3,000 sections with a small number of disectors, we feel the first scenario is likely. The direct examination of cetacean brains using our method will show whether or not the scaling rules for artiodactyl brains do extend to Cetartiodactyla as a whole. Such a study will also illuminate what happened in evolution when modern cetaceans branched off from the common ancestor with modern artiodactyls.

In the meantime, the three billion neurons that we predict to compose the cerebral cortex of the pilot whale is about one-fifth of the number of neurons found in the human cerebral cortex (Azevedo et al., 2009), even though the pilot whale cortex is twice larger than the human cortex. We predict, therefore, that even the largest cetacean cerebral cortex still has fewer neurons than the human cerebral cortex, supporting our hypothesis that the remarkable cognitive abilities of the human brain compared to even larger brains are related to the remarkable number of neurons in its brain, despite its modest size (Herculano-Houzel, 2009).

## Conflict of Interest Statement

The Associate Editor Chet C. Sherwood declares that, despite having collaborated with one of the authors (Paul R. Manger), the review process was handled objectively. José Maldonado is the representative in Latin America for MBF Bioscience, the company that markets the NeuroLucida software employed in this study. The other authors declare that the research was conducted in the absence of any commercial or financial relationships that could be construed as a potential conflict of interest.
