# Corrigendum: Cellular scaling rules for the brain of Artiodactyla include a highly folded cortex with few neurons

**DOI:** 10.3389/fnana.2015.00039

**Published:** 2015-03-26

**Authors:** Rodrigo S. Kazu, José Maldonado, Bruno Mota, Paul R. Manger, Suzana Herculano-Houzel

**Affiliations:** ^1^Instituto de Ciências Biomédicas, Universidade Federal do Rio de JaneiroRio de Janeiro, Brazil; ^2^Instituto Nacional de Neurociência Translacional, CNPq/MCTSão Paulo, Brazil; ^3^Microbrightfield BiosciencesBurlington, VT, USA; ^4^Instituto de Física, Universidade Federal do Rio de JaneiroRio de Janeiro, Brazil; ^5^School of Anatomical Sciences, University of the WitwatersrandJohannesburg, South Africa

**Keywords:** evolution, brain size, number of neurons, gyrification, cell size

It has come to our attention that some of the data presented in Table 1, on the mass, numbers of neurons and densities of brain structures in *Artiodactyla*, required minor corrections. Specifically, while the legend informed that total values for the cerebral cortex included the hippocampus (as in our previous studies), we recently realized that values for the hippocampus had in four cases been included in the rest of brain, not cerebral cortex, in Table 1, and had failed to be included for *Damaliscus*. There were a few other minor mistakes in the table that are now also corrected in Table 1 below.

**Table 1:** **Cellular composition of Artiodactyla brains**.

	***Sus scrofa domesticus***	***Antidorcas marsupialis***	***Damaliscus dorcas phillipsi***	***Tragelaphus stripceros***	***Giraffa camelopardalis***
M_BD_, kg	~100	25	60	218	470
M_BR_, g	64.180	106.074	154.718	306.860	537.218
M_CxT_, g	42.202	68.806	111.310	213.370	398.808
M_HP_, g	1.928	3.434	2.266	10.936	7.486
M_CB_, g	8.128	11.458	13.402	31.776	67.730
M_RoB_, g	13.850	25.810	30.006	61.716	70.680
M_D+BG_, g	6.728	12.194	n.a.	30.418	33.322
M_MES_, g	2.338	5.304	n.a.	12.902	15.928
M_P+M_, g	4.784	8.312	8.060	18.396	21.430
M_OB_, g	0.822	1.200	n.a.	5.546	2.052
N_BR_	2.22 × 10^9^	2.72 × 10^9^	3.06 × 10^9^	4.91 × 10^9^	10.75 × 10^9^
N_CxT_	307.08 × 10^6^	396.90 × 10^6^	570.67 × 10^6^	762.57 × 10^6^	1.73 × 10^9^
N_GM_	207.75 × 10^6^	293.77 × 10^6^	361.95 × 10^6^	596.21 × 10^6^	1.33 × 10^9^
N_HP_	12.91 × 10^6^	20.48 × 10^6^	22.09 × 10^6^	28.36 × 10^6^	58.59 × 10^6^
N_CB_	1.86 × 10^9^	2.26 × 10^9^	2.40 × 10^9^	4.04 × 10^9^	8.88 × 10^9^
N_RoB_	58.71 × 10^6^	70.48 × 10^6^	86.43 × 10^6^	106.59 × 10^6^	142.70 × 10^6^
N_D+BG_	34.40 × 10^6^	40.12 × 10^6^	n.a.	58.88 × 10^6^	68.63 × 10^6^
N_MES_	12.43 × 10^6^	7.52 × 10^6^	n.a.	26.07 × 10^6^	26.63 × 10^6^
N_P+M_	11.88 × 10^6^	22.84 × 10^6^	20.72 × 10^6^	21.64 × 10^6^	47.72 × 10^6^
N_OB_	9.20 × 10^6^	16.00 × 10^6^	n.a.	38.33 × 10^6^	24.68 × 10^6^
DN_CxT_	7276	5768	5127	3574	4339
DN_GM_	7375	6684	5142	4644	5882
DN_HP_	6695	5965	9750	2594	8435
DN_CB_	228,632	196,999	179,206	127,218	131,080
DN_RoB_	4238	2731	2880	1727	2019
DN_D+BG_	5113	3290	n.a.	1936	2060
DN_MES_	5317	1418	n.a.	2594	1672
DN_P+M_	2483	2748	2570	1176	2227
DN_OB_	11,187	13,332	n.a.	6912	12,026
O/N_BR_	2.111	2.170	3.054	3.456	3.526
O/N_CXT_	10.585	10.396	11.851	16.133	15.900
O/N_GM_	8.544	7.239	8.356	8.754	7.763
O/N_HP_	10.334	10.111	9.246	17.868	10.628
O/N_CB_	0.188	0.207	0.184	0.313	0.622
O/N_RoB_	18.682	18.710	24.718	31.980	34.190
O/N_D+BG_	17.779	19.408	n.a.	31.841	38.841
O/N_MES_	15.667	30.250	n.a.	30.546	41.017
O/N_P+M_	24.452	13.706	22.810	34.088	23.483
O/N_OB_	8.434	6.576	n.a.	8.523	9.417

Cellular composition of the five artiodactyl species. M, mass of body (M_BD_) or brain structure; N, number of neurons; DN, neuronal density (in neurons/mg); O/N, ratio between numbers of other (non-neuronal) cells and neurons. BR, whole brain (excluding the olfactory bulb); CXT, whole cerebral cortex (gray matter, white matter and hippocampus); GM, gray matter of the cerebral cortex; HP, hippocampus; CB, cerebellum; RoB, rest of brain (the sum of diencephalon + basal ganglia, mesencephalon, and pons + medulla); D + BG, diencephalon + basal ganglia; MES, mesencephalon; P + M, pons + medulla; OB, olfactory bulb. All values refer to the two hemispheres together.

While these corrections do not modify in any way the conclusions of the paper, some of the power exponents reported were influenced in minor, non-significant ways. Those corrected power exponents are also provided below.

**Corrections in text:**

p. 4 – Brain mass varies 8.4-fold, number of brain neurons varies 4.8-fold.

**Corrected relationships and power functions:**

p. 4, Figure 3A – Brain mass increases as a power function of body mass with a small exponent of 0.548 ± 0.038 (*p* = 0.0048).

p. 4, Figure 3C – The total number of brain neurons increases as a power function of body mass with an exponent of 0.448 ± 0.115 (*p* = 0.0598).

p. 5 – The relative mass of the rest of brain does not decrease significantly with increasing brain mass (Spearman correlation, ρ = −0.800, *p* = 0.1041).

p. 7 – The cerebral cortex has only 15.7 ± 0.8% of all brain neurons, despite representing 69.5 ± 1.8% of brain mass, and the rest of brain, which accounts for 19.6 ± 1.8% of brain mass, has only 2.3 ± 0.3% of all brain neurons.

p. 7, Figure 4A – Total brain mass varies as a power function of its number of neurons with an exponent of 1.288 ± 0.215 (*r*^2^ = 0.923, *p* = 0.0093).

p. 7, Figure 4B – The relationship between the mass of the cerebral cortex and its number of neurons has an exponent of 1.303 ± 0.154 (*p* = 0.0035) including the giraffe, and 1.721 ± 0.123 (*r*^2^ = 0.990, *p* = 0.0051) excluding the giraffe.

p. 7, Figure 4D – The mass of the rest of brain scales as a power function of its number of neurons across artiodactyls with an exponent of 1.850 ± 0.303 (*r*^2^ = 0.925, *p* = 0.0089).

p. 7, Figure 5A – The relationship between mass of each brain structure (cerebral cortex, cerebellum and rest of brain) and number of other (non-neuronal) cells can be described as a single power function of exponent 0.859 ± 0.047 (*p* < 0.0001).

p. 7, Figure 5B – Whole brain mass varies as a similar function of numbers of other cells across artiodactyls (exponent 0.986 ± 0.089, *p* = 0.0016) (…) and all clades together (exponent, 1.040 ± 0.020, *p* < 0.0001).

p. 8 – Neuronal density in the cerebral cortex (gray + white matter + hippocampus) varies between 3574 neurons/mg in the greater kudu to 7276 neurons/mg in the pig (…) and in the rest of brain, from 1727 neurons/mg in the greater kudu to 4238 neurons/mg in the pig. p. 9, Figure 6A – Neuronal density in the artiodactyl cerebral cortex (minus the giraffe) decreases with increasing cortical mass, as a power function of exponent −0.425 ± 0.041 (*p* = 0.0093).

p. 9, Figure 6A – In the rest of brain, neuronal density also decreases significantly as a power function of increasing structure mass (exponent, −0.500 ± 0.082, *p* = 0.0089).

p. 10, Figure 7 – The O/N ratio varies between 0.184 (in the blesbok cerebellum) and 34.190 (in the giraffe rest of brain). The O/N ratio varies within the cortical gray matter alone between 7.2 and 8.8 across species.

p. 10, Figure 7B – The O/N ratio varies as a common power function of neuronal density across all artiodactyl structures with an exponent of −1.087 ± 0.032 (*p* < 0.0001).

p. 10 – The addition of artiodactyl structures does not change the exponent significantly (−0.935 ± 0.021, *p* < 0.0001).

p. 11 – N/A is 2–6 times smaller in artiodactyls (19,902 ± 1253 neurons/mm^2^) than in primates.

p. 11, Figure 8C – Cortical surface area increases with numbers of neurons raised to an exponent of 1.362 ± 0.094 across artiodactyls (*p* = 0.0047).

p. 11 – Gray matter thickness increases with number of cortical neurons raised to the power of 0.630 ± 0.089 in artiodactyls (minus the giraffe; *p* = 0.0192).

p. 13 – Predictions for cetaceans: The prediction is given by the equation *N*_CXT_ = *e*^17.168±0.053^. *M*^0.633±0.024^_CXT_.

p. 14 – Using the cortical volume given (…) we predict the cerebral cortex (…) to be composed of 1.14, 1.99, 2.44, and 3.56 billion neurons, respectively.

## Conflict of interest statement

The authors declare that the research was conducted in the absence of any commercial or financial relationships that could be construed as a potential conflict of interest.

